# Long range inter-chromosomal interaction of *Oct4* distal enhancer loci regulates ESCs pluripotency

**DOI:** 10.1038/s41420-023-01363-8

**Published:** 2023-02-13

**Authors:** Byoung-San Moon, David Huang, Fan Gao, Mingyang Cai, Guochang Lyu, Lei Zhang, Jun Chen, Wange Lu

**Affiliations:** 1 Department of Biotechnology, Chonnam National University, Yeosu, 59626 Korea; 2 Department of Stem Cell Biology and Regenerative Medicine, Broad Center for Regenerative Medicine and Stem Cell Research, Keck School of Medicine, University of Southern California, Los Angeles, CA 90033 USA; 3 State Key Laboratory of Medicinal Chemical Biology and College of Life Sciences, Nankai University, 94 Weijin Road, 300071 Tianjin, China

**Keywords:** Nuclear organization, Embryonic stem cells, Nuclear organization

## Abstract

Nuclear architecture underlies the transcriptional programs within the cell to establish cell identity. As previously demonstrated, long-range chromatin interactions of the *Oct4* distal enhancer (DE) are correlated with active transcription in naïve state embryonic stem cells. Here, we identify and characterize extreme long-range interactions of the *Oct4* DE through a novel CRISPR labeling technique we developed and chromosome conformation capture to identify lethal giant larvae 2 (*Llgl2*) and growth factor receptor-bound protein 7 (*Grb7*) as putative functional interacting target genes in different chromosomes. We show that the *Oct4* DE directly regulates expression of *Llgl2* and *Grb7* in addition to *Oct4*. Expression of *Llgl2* and *Grb7* closely correlates with the pluripotent state, where knock down of either result in loss of pluripotency, and overexpression enhances somatic cell reprogramming. We demonstrated that biologically important interactions of the *Oct4* DE can occur at extreme distances that are necessary for the maintenance of the pluripotent state.

## Introduction

Genome architecture has emerged as an important cell type-specific epigenetic signature with roles in regulating eukaryotic cell identity and function. As cells need to regulate gene expression temporally in a cell-type and lineage-specific manner, spatially restricting chromatin positioning may provide an additional layer of regulation that can be highly flexible and responsive to external cues. Interestingly, however, much of the overall nuclear architecture remains mostly stable and self-contained in recently described topological associating domains (TADs), while only a small fraction of dynamic chromatin has been observed to loop outside of their nuclear compartment [1, 2] Considered to be a non-emergent phenomenon, active long-range chromatin looping occurs to physically bring cis-acting regulatory elements like promoters and distally located enhancers together through binding of specific transcription factors and chromatin remodeling complexes [3, 4]. Distinct patterns of enhancer-promoter interactions help define the regulatory landscape within specific cell types.

Many studies examining enhancers dynamics have revealed several interesting properties: enhancers can regulate multiple genes simultaneously and create clusters of co-regulated genes in an active chromatin hub [5–8], do not always regulate their most linearly proximal promoters [9], and many have been observed interacting with promoters located hundreds of kilobases away or even on other chromosomes [4, 10]. Recent description of “super enhancer” regions are able to coordinate cell identity and function through lineage-specific chromatin looping activity regulated through recruitment of the Mediator complex and cell type-specific transcription factors [11, 12]. Characterizing the activity of important super enhancers can inform how cell type-specific gene expression networks are established and maintained.

Various techniques have been developed for investigating genome organization, with chromosome conformation capture (3C) derived techniques as the most widely used methods; but 3C assays are constrained by certain limitations. Based on the principle of biochemically fixing the spatial positioning of the chromatin, interacting loci can be detected through measuring the frequency of ligated bait-target junction fragments [13]. However, artificial fixation of the chromatin can lead to interaction artifacts that do not represent truly interacting loci [14–16]. In addition, intrinsic biases of the method tend to over represent the most proximally located fragments to the bait viewpoint, which can obscure rare and transient or long distance interactions [17]. Such issues in 3C data have resulted in discrepancies when validating interactions using other techniques such as DNA fluorescence in situ hybridization [18].

A parallel technique used to identify protein-binding at DNA loci in vivo called DamID potentially overcomes some of these challenges. By fusing prokaryote unique DNA adenine methyltransferase (Dam) to a DNA binding protein of interest, proximity labeling through adenine methylation of GATC sequences at interacting sites can occur within the vicinity of protein-binding sites in live cells independent of distance [19–22]. Labeled sites can then be assayed directly by sequential digestion with DpnI and DpnII restriction enzymes that are methylation sensitive to the common GATC recognition motif to provide an unmodified interaction profile stemming from a locus of interest. DamID has been applied in many cell types and organisms to reveal binding profiles for a multitude of transcription factors, insulators, and chromatin-modifying proteins within many biological contexts [23]. As Dam is able to methylate both cis and trans loci, site-specific targeting of Dam can produce 3C equivalent interaction profiles [19]. However, previous studies required insertion of the yeast transcription factor GAL4 sequence to the site of interest to guide the Dam-GAL4 fusion protein, making study of other loci a laborious and low throughput process [19, 24]. Fortunately, the advent of CRISPR-Cas9 technology has simplified the challenge of site-specific targeting which we demonstrate in our study.

As the focus of our study of embryonic stem cell (ESC) pluripotency, the *Oct4* super enhancer region is an important regulator of the pluripotent stem cell state. Three cis-regulatory regions upstream of the Pou5f1 gene transcription start site (TSS) have previously been characterized: the proximal promoter (PP), the proximal enhancer (PE), and distal enhancer (DE) [25, 26]. The DE and PE are utilized in a cell-type specific manner in pluripotent cells to regulate *Oct4* expression, where the DE controls active transcription of *Oct4* in naïve state pluripotency while the PE is active in primed state pluripotency [27, 28]. Two distinct regulatory elements within the DE have also been identified, conserved regions CR3 and CR4, that are shared across different species and contain binding sites for many transcription factors with positive and negative regulatory roles on *Oct4* expression [25, 26, 29, 30]. In studying the activity of the *Oct4* locus in naïve state ESCs, our previous work has shown that endogenous *Oct4* transcription occurs concurrently with long-range chromatin interactions at the *Oct4* locus and binding of looping factors at the DE [31, 32]. Concomitant to loop formation, chromatin remodeling complexes mediator and cohesin are recruited by Klf4 and subsequently leads to RNAPII recruitment. With these observations, we predicted that other putative looping targets of the DE should exist and remain to be characterized.

In our study, we developed a novel hybrid method combining CRISPR and DamID to investigate long-range looping interactions of the *Oct4* DE and identify new direct participants of the pluripotency gene network. Through the use of this new method and traditional DNA fluorescence in situ hybridization (FISH) and chromosome conformation capture (3C) techniques, we observe the *Oct4* DE interacting with two genes located on chromosome 11, lethal giant larvae 2, *Llgl2*, and growth factor receptor bound protein 7, *Grb7*. We show that these interactions are directly regulated by the distal enhancer via the critical CR4 region, where deletion of this region correlated with loss of target gene expression and pluripotency despite restoration of *Oct4* levels. We also characterize the functional role of *Llgl2* and *Grb7* through loss of function and gain of function assays and confirm that both participate in maintaining pluripotency in ESCs. These results demonstrate previously unexplored interchromosome interactions by the *Oct4* DE that directly regulate the expression of pluripotency-related genes.

## Results

### 4C-seq and CRISPR DamID seq reveals long-range interactions of the *Oct4* distal enhancer

Previously, we employed circular chromosome conformation capture and sequencing (4C-seq) to map the interactome in E14 mouse embryonic stem cells [32]. Designating the DE bait region ~2 kb upstream of the mouse Pou5F1 gene transcription start site, we observed about 15% of captured reads were interchromosomal interactions shared between two biological replicates. Regions positively identified in 4C-seq colocalized more frequently with the *Oct4* locus than non-4C regions.

**Fig. 1:**
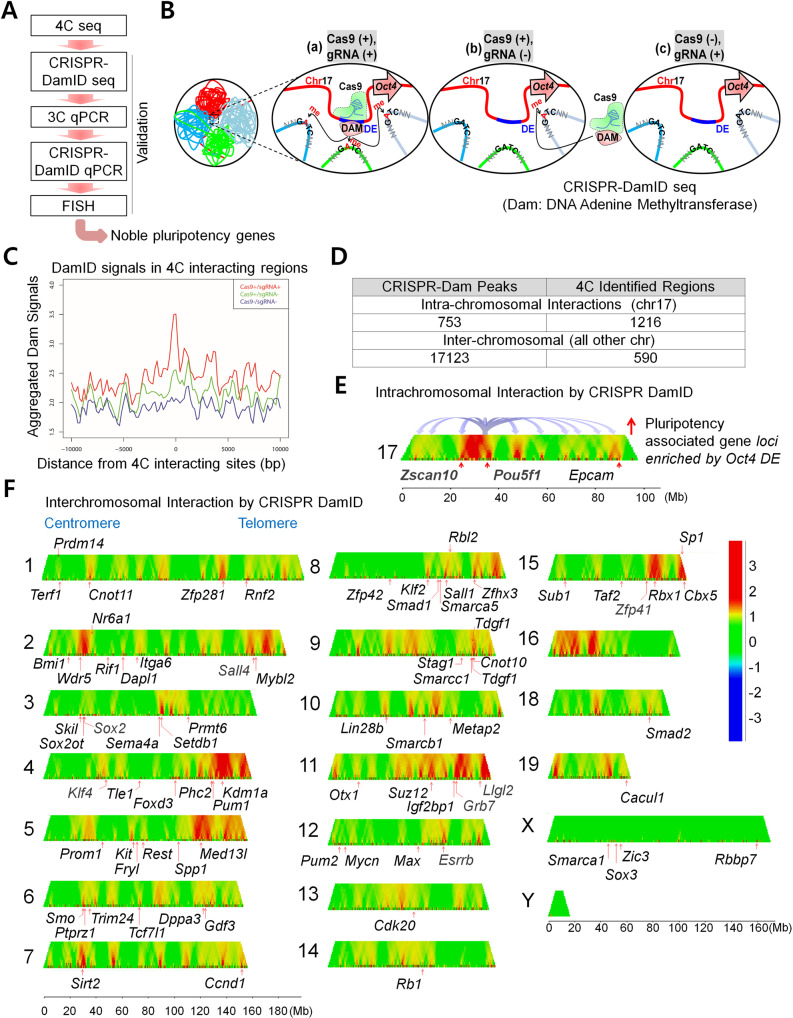
The *Oct4* distal enhancer locus binds to promoter regions of ESC pluripotency-associated gene loci intra- and inter-chromosomally. **A** Schematic diagram illustrating summary of our study. Previous 4C-seq results [32] is further validated by the CRISPR-DamID hybrid system. 3C qPCR, CRISPR-DamID qPCR and DNA FISH analysis are used for validation of 4C-seq and CRISPR-DamID seq results; **B** Schematic of the CRISPR-Dam labeling assay. The doxycycline inducible Nm-dCas9-Dam fusion protein is targeted by guide RNA to the *Oct4 DE* locus and proximity labeling occurs at GATC sites within the vicinity. Control samples not containing small guide RNA with Cas9-Dam expression (Cas9+/gRNA−) and samples with guide RNA but without induction of Cas9-Dam (Cas9−/gRNA+) are included to normalize potential nonspecific labeling; **C** Overlap of CRIPSR-Dam peaks with 4C-seq identified regions around 4C sites. Cas9+/gRNA+ 6-h peaks are enriched compared to Cas9+/gRNA− and Cas9-/gRNA+ samples; **D** Summary of CRISPR-Dam peaks located on chromosome 17 (intrachromosomal interactions) and all other chromosomes (interchromosomal interactions). Previously identified 4C-seq regions are also summarized by genome distribution; **E**, **F** Domainogram representation of CRISPR-Dam coverage on chromosome 17 and other chromosomes. Pluripotency-related genes are labeled in the regions indicated.

To pin down the more specific interacting loci and identify putative gene targets located within these regions, we performed cross-validation of 4C-seq results through CRISPR-DamID hybrid assay (Fig. 1A). By fusing *Escherichia coli* DNA adenine methyltransferase (DAM) to a catalytically dead *Neisseria meningitides* Cas9, we constructed a targetable live cell labeling system combining the principles of CRISPR-Cas9 and DamID to track long-range chromatin interactions at the *Oct4* DE (Fig. 1B). The Cas9 protein targets specific loci of interest while the Dam domain labels interacting loci by methylating adenines at proximally located GATC sites. The type II CRISPR system in *N. meningitidis* was chosen over the more commonly used *Streptococcus pyogenes* CRISPR-Cas9 due to the longer protospacer adjacent motif (PAM) sequence utilized for guide RNA targeting, which could allow for comparatively more specific locus recognition [33, 34]. To control Dam activity, we selected a doxycycline-inducible promoter to drive Cas9-Dam expression in E14 mouse embryonic stem cells. We confirmed the expression of our FLAG-tagged NmCas9-Dam fusion protein, which appeared by 6 h after the addition of doxycycline by Western Blot and qPCR (Supplementary Fig. 1A, B and Original data file 2) as well as effect of two validated *Oct4* DE targeting Nm-gRNAs by qPCR (Supplementary Fig. 1C). For normalization of background signals, we also constructed E14 cell lines containing inducible NmCas9-Dam without Nm-gRNA expression (Cas9+/gRNA−) and included uninduced samples as negative controls (Cas9−/gRNA+). CRISPR-Dam integrated cells were induced with doxycycline for 6 h before total genomic DNA was harvested and prepared using the dual enzyme digestion process (see Methods) for library preparation and sequencing. Sequencing reads were aligned to the mm9 genome. We called out significant peaks using HOMER, which found nearly 17,000 unique peaks in our Cas9+/gRNA+ samples and ~8900 unique peaks in our Cas9+/gRNA− sample (Supplementary Table 4). Only about 5% of unique peaks were shared between our gRNA+ and gRNA- samples. Samples expressing Cas9-Dam and sgRNA had signals overlapping well with 4C-identified sites (Fig. 1C). The numbers of CRISPR-Dam peaks of intra-chromosome interactions on chromosome 17 identified by Cas9-Dam was about half of 4C-seq identified sites. Moreover, as much as 30 folds of inter-chromosome interactions were identified in the CRISPR-Dam samples compared with 4C-seq (Fig. 1D and Supplementary Table 4). Domainogram shows *Oct4* DE interaction sites are not distributed evenly throughout the genome, instead they are enriched in some “hot” regions of some chromosomes (Fig. 1E, F). In addition, *Oct4* DE interacts with many known pluripotency gene loci on chromosome 17 such as *Zscan10*, *Pou5f1* as well as on other chromosomes such as *Klf4, Sox2, Nanog*, *Sall4*, *Esrrb, and Zfp41* (also known as *Rex1)* (Fig. 1E, D and Supplementary Fig. 2). Furthermore, we observed close alignment of the CRISPR-Dam peaks with active histone marks such as histone marks for active enhancers H3K27ac, poised enhancers H3K4me1, and active promoters H3K4me3 (Supplementary Fig. 2). We also observed that our CRISPR-Dam peaks lie at architectural protein CTCF and cohesin binding sites, suggesting *Oct4* DE tends to interact with chromatin loci with active gene transcription or chromatin looping.

### *Oct4* distal enhancer interacts with *Llgl2* and *Grb7* loci on chromosome 11

**Fig. 2:**
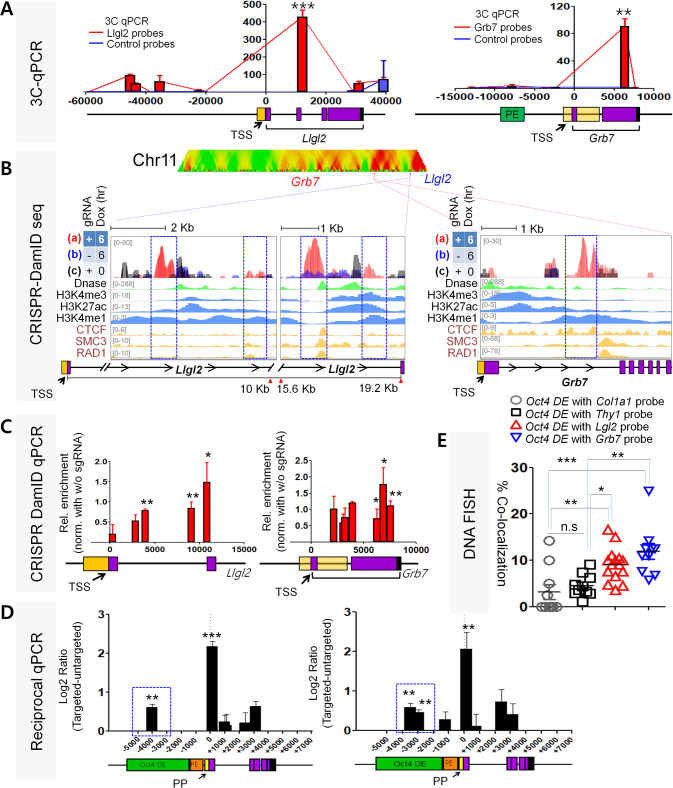
The *Oct4* distal enhancer locus interacts with promoter regions of *Llgl2* and *Grb7*. **A** Chromosome Conformation Capture (3C)-qPCR results for interchromosomal interaction frequency between the *Oct4 DE* locus on chromosome 17 and *Llgl2* and *Grb7* regions on chromosome 11. Same triplicate HindIII cut libraries used as templates and HindIII sites assayed as in PCR. Frequencies normalized to an internal control. Error bars indicate standard deviation for biological triplicates; **B** (Upper) Domainogram representation of CRISPR-Dam coverage on chromosome 17. (Lower) Profile of CRISPR-Dam peaks at Llgl2 and Grb7 loci. ENCODE tracks are included for histone modifications H3K27ac, H3K4me1, and H3K4me3, DNA accessibility represented by DNaseI hypersensitivity peaks, and architectural protein binding sites for CTCF, SMC3, and RAD21. Boxed regions (in blue) highlight CRISPR-Dam peaks aligned with active histone marks. Distinct regions flanked by architectural proteins binding sites suggest these regions may be looped out (red and green highlight); **C** CRISPR-Dam qPCR querying individual GATC sites around identified sequencing peaks at Llgl2 and Grb7 loci. Bars correspond to individual GATC sites. Values calculated from triplicate samples normalized with no guide RNA and no doxycycline-induced samples; **D** CRISPR-Dam qPCR detecting reciprocal interaction from Llgl2 and Grb7 identified CRISPR-Dam peaks. Bars correspond to individual GATC sites. Values calculated from triplicate samples normalized with no guide RNA and no doxycycline-induced samples. Blue box highlights interaction signals at the Oct4 DE; **E** Colocalization frequency between *Oct4 DE* region and *Llgl2*, *Grb7*, *Thy1*, and *Col1A1* regions per field of view; In panels **A**, **C**, **D**, **E**, error bars indicate mean ± SD. All data were compared by one-way ANOVA with Tukey’s post hoc test. (*p < 0.01, **p < 0.001, ***p < 0.0001).

Previous 4C-seq and DNA FISH results suggested that *Oct4* locus showed high colocalization frequencies between *Oct4* locus and 143F14 and 474J5 loci at chromosome 11 [32]. After further analysis of the signals within the two regions of interest on chromosome 11, we employed high resolution 3C assays to probe the region around our gene targets. 3C-PCR and 3C-qPCR primers were designed to tile ~10 kb around the gene clusters containing lethal giant larvae 2, *Llgl2*, and growth receptor bound protein 7, *Grb7* (Supplementary Fig. 3). The 6 bp cutting enzyme HindIII was initially chosen as there was only a single HindIII site within the *Oct4* DE we could designate as our bait. Duplicate 3C libraries were each prepared with 1 × 10^7^ E14 cells and 3C-PCR was performed to detect ligated bait-target fragments. Resultant band intensities were normalized to an internal reference site and corresponding band intensities from control BAC libraries also prepared with HindIII (Supplementary Table 5). We found 3C interaction signals at the gene cluster containing promoters of *Llgl2* (Supplementary Fig. 3A–C) and a clear interaction signal at the *Grb7* locus (Supplementary Fig. 3D–F). We also performed 3C-qPCR analysis to confirm our results (Fig. 2A). These interactions were further validated by our CRISPR-DamID seq analysis. We observed distinct peaks located at gene body regions of *Llgl2* and *Grb7*, respectively (Fig. 2B). When aligned with ENCODE tracks for histone marks H3K27ac, H3K4me1, and H3K4me3, our CRISPR-Dam peaks nearly overlap with these active epigenetic marks(Fig. 2B). We were also able to see both *Llgl2* and *Grb7* loci bounded by CTCF binding and cohesin components SMC3 and RAD21 binding, which suggested these regions were looped out for potential interaction with the *Oct4* DE (Supplementary Fig. 4A–C). To confirm the sequencing results, we performed quantitative PCR by designing primer pairs to probe peak adjacent GATC sites located ~6 kb around the transcription start sites (TSS) of *Llgl2* and *Grb7*. The same CRISPR-Dam E14 cell lines were induced to express Cas9-Dam for 6 h before collection and digestion with only DpnII. qPCR probes tiling the gene loci showed correlating profiles with each enriched sequencing peak we had previously observed (Fig. 2C and Supplementary Table 6). Agreement of the results from sequencing and qPCR demonstrated that our CRISPR-Dam system could reproducibly identify interacting loci of *Oct4* DE in embryonic stem cells. As further confirmation of our CRISPR-Dam identified interaction results, we also performed reciprocal CRISPR-Dam experiments by targeting the Cas9-Dam to those peak regions in *Llgl2* and *Grb7*, followed by qPCR to detect CRISPR-Dam labeling of GATC sites at the *Oct4* locus. We observed interaction signals at the DE as well as the proximal promoter and gene body of *Oct4*, indicating that interactions between the *Oct4* DE and *Llgl2* and *Grb7* could be confirmed by our CRISPR-Dam technique (Fig. 2D). To visually verify the interactions in cells, we utilized DNA-FISH using hapten probes generated from selected bacterial artificial chromosomes encompassing our loci of interest. Probes targeted to *Llgl2* and *Grb7* regions showed significantly higher colocalization frequency with the *Oct4* locus compared to other regions, such as one containing a non-expressed, fibroblast marker gene *Thy1* and a different probe targeted to ubiquitously expressed gene *Col1A1* located near *Llgl2* and *Grb7* loci also on chromosome 11 (Fig. 2E and Supplementary Table 7) [35]. These observations along with the results from our CRISPR-Dam assay indicated that *Llgl2* and *Grb7* are candidate interacting gene targets of the *Oct4* DE.

### *Oct4* distal enhancer CR4 region directly regulates *Llgl2* and *Grb7*

While Oct4 protein has been extensively studied for its role in pluripotency, the role of the *Oct4* DE in naïve state pluripotent cells has not been fully explored. Dissection of the DE have revealed several critical conserved regions (CR) shared across species containing multiple transcription factor binding sites, with the CR4 region shown to be necessary for auto-regulation of *Oct4* expression by an Oct4-Sox2 complex [29, 30]. Insufficient *Oct4* expression directly leads to loss of pluripotency gene expression network and initiates differentiation gene expression [36]. From our CRISPR-Dam gRNA site and 3C bait regions designated at the HindIII site at the CR4 region, we hypothesized that looping interactions of the *Oct4* DE may be regulated by the CR4 region and be directly involved in activation of target gene expression.

**Fig. 3:**
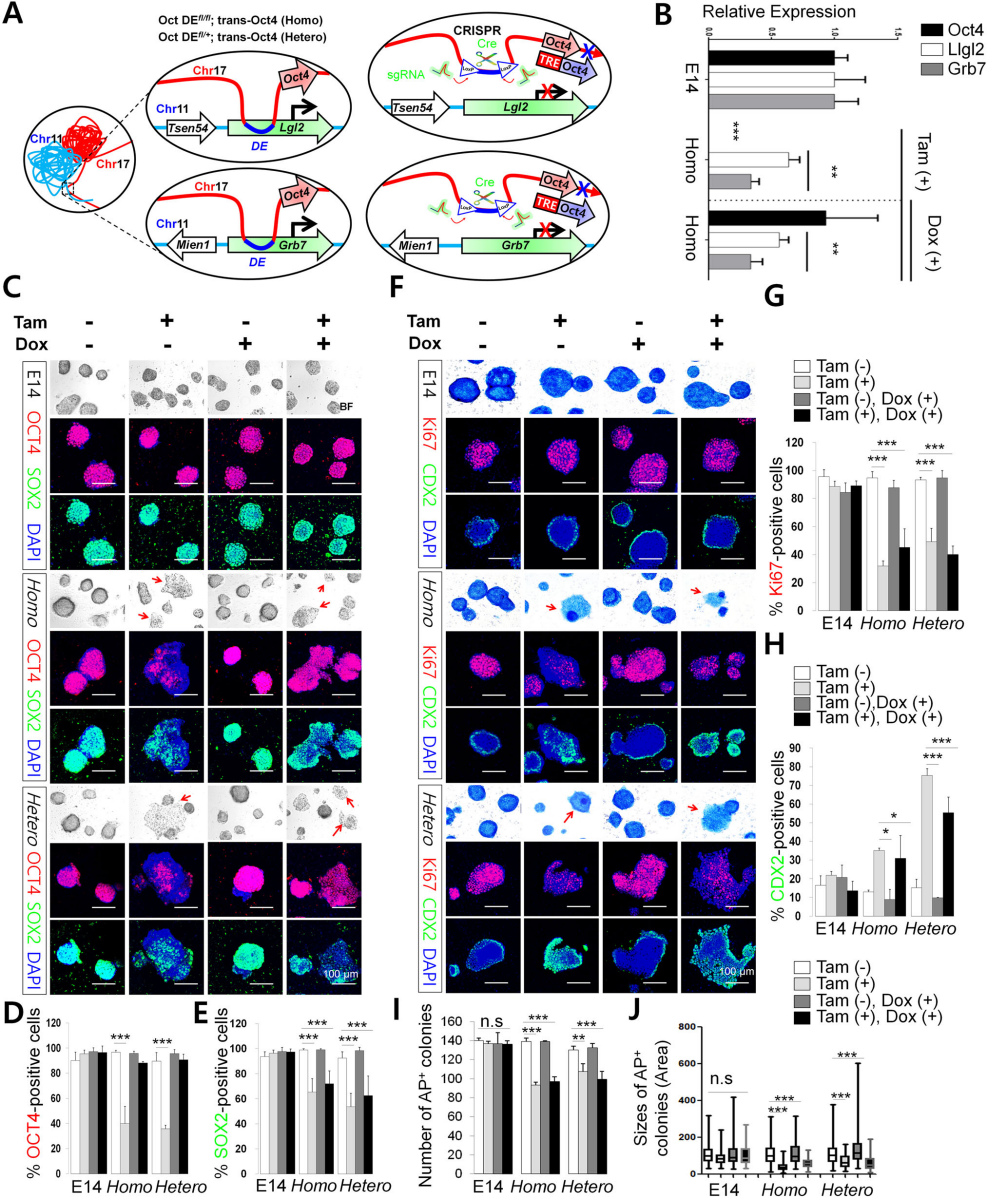
Oct4 distal enhancer CR4 region directly regulates *Llgl2* and *Grb7* expression as part of maintaining stem cell pluripotency and self-renewal. **A** Schematic of loxP sites insertion flanking CR4 region within the *Oct4* DE. Pairs of guide RNAs were designed with corresponding oligonucleotide repair templates containing loxP sites and modified PAM sequences. Doxycycline inducible transgenic *Oct4* mRNA and tamoxifen-inducible CreERT2 were also inserted in the cells; **B** qPCR comparison of wild type (+/+) and bi-allelic (−/−) loxP inserted ESC clones for *Oct4*, *Llgl2*, and *Grb7* expression after tamoxifen and/or doxycycline treatment; **C** Immunofluorescence staining of Oct4 and Sox2 in Oct4 DE+/+,+/−, and −/− cells. Red arrows specify differentiating cells with flattened morphology; **D**, **E** Quantification of positive immunofluorescent wild type E14, Oct4 DE homozygous (−/−) and heterozygous (+/-) deleted cells for Oct4 and Sox2 expression; **F** Immunofluorescence staining of Cdx2, Ki67, and alkaline phosphatase in Oct4 DE+/+,+/−, and −/− cells. Red arrows specify differentiating cells with flattened morphology; **G**, **H** Quantification of positive immunofluorescent wild type E14, *Oct4* DE homozygous (−/−) and heterozygous (+/−) deleted cells for Cdx2 and Ki67 expression. Double positive cells are also quantified; **I** Quantification of alkaline phosphatase positive Oct4 DE+/+,+/−, and −/− cells colonies; **J** Colony sizes of AP-stained colonies by pixel area; In panels **C**, **F** representative images shown at 100 μm scale; In panels **B**, **D**, **E**, **G**, **H**, **I**, **J** data plotted are mean ± SD for triplicate samples and were compared by one-way ANOVA with Tukey’s post hoc test. (*p < 0.01, **p < 0.001, ***p < 0.0001).

To determine if the *Oct4* DE plays a direct role in regulating *Grb7* and *Llgl2* gene expression in addition to *Oct4* gene expression, we designed doxycycline-inducible transgenic *Oct4* E14 cell lines in which an independently inducible Cre-loxP system can conditionally knock out the DE while maintaining total *Oct4* levels (Fig. 3A and Supplementary Table S8). Mono-allelic and bi-allelic *Oct4* DE deletion cell lines, −/− and +/− respectively, were isolated and clonally expanded for comparison with wild-type+/+ cells. Tamoxifen treatment led to efficient deletion of *Oct4* DE (Supplementary Fig. 5A), while doxycycline treatment induced transgenic *Oct4* expression, which supplemented endogenous *Oct4* expression (Supplementary Fig. 5B). qPCR was performed to measure total *Oct4* expression as well as *Llgl2* and *Grb7* expression after treatment with tamoxifen or tamoxifen and doxycycline (Fig. 3B). In the tamoxifen only treatment condition, *Oct4* gene expression was almost completely abolished in the *Oct4* DE −/− lines, confirming that the *Oct4* DE is required for *Oct4* expression. In addition, both *Llgl2* and *Grb7* expression was reduced in the −/− cell line, suggesting that they may be regulated by the *Oct4* DE. To rule out the possibility that the reduction of *Llgl2* and *Grb7* expression is an indirect effect of reduced *Oct4* gene expression, we measured *Llgl2* and *Grb7* levels where *Oct4* gene expression was restored to wild-type levels by transgenic *Oct4* induction through the addition of doxycycline. Neither *Llgl2* nor *Grb7* expression was rescued after doxycycline treatment in the −/− cell line in which *Oct4* expression is restored to a similar level to wild-type cells (Fig. 3B), indicating a direct role of the *Oct4* DE on *Llgl2* and *Grb7* expression.

Stemness and differentiation of these treated cells were then examined using immunofluorescence staining and alkaline phosphatase (AP) staining. Upon tamoxifen treatment, OCT4 staining was reduced in heterozygous and homozygous deletion cells as expected, so were staining of pluripotency marker SOX2 and cell proliferation marker Ki67 (Fig. 3C–G), suggesting that *Oct4* DE deletion cell exhibited reduced self-renewal and proliferation. Instead, these cells exhibited differentiation phenotype as indicated by the trophectoderm marker CDX2 (Fig. 3F–H). OCT4 levels could be restored with doxycycline treatment in the tamoxifen-treated homo-, or heterozygous deletion cells (Fig. 3B–D). Interesting, restoration of Oct4 could not restore the stemness of these cells as demonstrated by immunostaining of SOX2 marker (Fig. 3C, D). These treated wild-type, heterozygous, and homozygous cell lines were also stained with AP. Cells treated with tamoxifen only or tamoxifen and doxycycline showed reduced numbers of AP positive cells (Fig. 3F, I and Supplementary Fig. 6) and reduced colony size (Fig. 3J), which supports our observation that pluripotency and self-renewal is perturbed with the deletion of one or both alleles *Oct4* DE despite even after restoring OCT4 expression. These experiments show that *Oct4* DE regulates stem cell self-renewal through regulating expression of genes in addition to Oct4.

To identify all other genes are directly regulated by the *Oct4* DE, we performed RNA-seq on our *Oct4* DE (−/−) deleted cells in tamoxifen only and tamoxifen and doxycycline conditions and overlapped the resulting global gene expression profiles with our previously identified CRISPR-Dam peaks (Supplemental Data 1, 2). We observed in the tamoxifen only condition cells 442 downregulated genes that consisted of many known pluripotency-related genes, including our target genes *Llgl2* and *Grb7* (Supplementary Fig. 7A–C). Many of the same pluripotency-related genes were also found in the 369 genes downregulated in the tamoxifen and doxycycline condition cells (Supplementary Fig. 7D–F). This further supported our observation that the *Oct4* DE may regulate genes throughout the genome that are involved in maintenance of pluripotency and self-renewal, including our newly identified candidates *Llgl2* and *Grb7*.

### *Llgl2* and *Grb7* knockdown correlates to disrupted pluripotency state

**Fig. 4:**
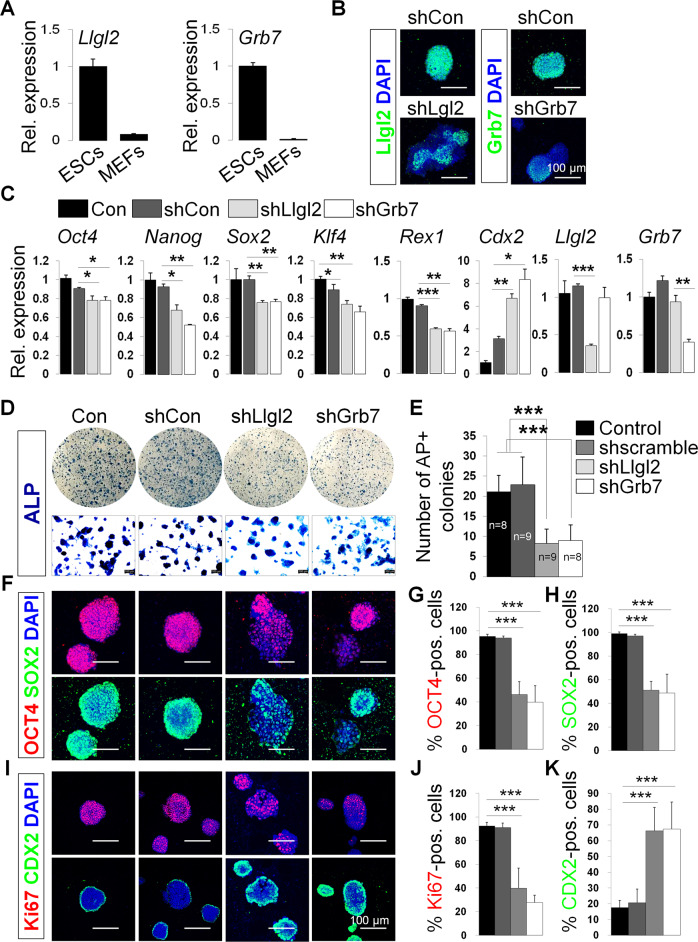
Knock down of Llgl2 and Grb7 abrogates mESC pluripotency. **A** qPCR on ESCs and MEFs for endogenous *Llgl2* and *Grb7* expression; **B** Representative images of Llgl2 and Grb7 immunofluorescence in ESCs after knocking down by shRNAs; **C** qPCR measuring pluripotency gene (*Oct4*, *Nanog*, *Sox2*, *Klf4*, *Rex1*) expression after knocking down Llgl2 and Grb7 compared to control knock down and wild-type E14 ESCs. Cdx2, a marker for trophectoderm, is also shown; **D** AP+ staining ESC colonies after knocking down by shRNAs for Llgl2 or Grb7 or control compared with wildtype E14 control colonies. **E** Quantification of AP+ staining ESC colonies per area (mm^2^); **F** Immunofluorescence staining of OCT4 and Sox2 in shRNA knock down cells; **G**, **H** Quantification of positive immunofluorescent knock down cells for Oct4 and Sox2 expression; **I** Immunofluorescence staining of Cdx2 and Ki67 in shRNA knock down cells; **J**, **K** Quantification of positive immunofluorescent knock down cells for Cdx2 and Ki67 expression. In panels **A**, data were compared by unpaired *t* tests (***p < 0.0001). In panels **B**, **F**, **I**, representative images shown at a 100μm scale. In panel **D**, representative images are shown at 40X scale. In panels **E**, **G**, **H**, **J**, **K**, data plotted are mean ± SD for triplicate samples and were compared by one-way ANOVA with Tukey’s post hoc test. (*p < 0.01, **p < 0.001, ***p < 0.0001).

As newly identified direct targets of the *Oct4* DE, we examined the functional role of *Llgl2* and *Grb7* in pluripotent cells. Comparing the expression of both genes in ESCs and non-pluripotent mouse embryonic fibroblasts, we observed much higher expression of these genes in pluripotent cells, suggesting that these genes may play a role in pluripotency (Fig. 4A). We then employed a shRNA pooling strategy to deplete the mRNA of each gene in E14 ESCs (Supplementary Table 2). Cells were incubated for 2 days after lipofection with shRNA plasmids targeting either *Llgl2* or *Grb7* before staining. We confirmed knockdown of endogenous *Llgl2* and *Grb7* in ESCs by immunofluorescence and qPCR (Fig. 4B, C). Expression of *Llgl2* and *Grb7* decreased by 70% and 75%, respectively upon shRNA knockdown (Fig. 4C). We also observed reduced expression of key pluripotency genes including *Oct4, Nanog, Sox2, Klf4*, and *Rex1* and upregulation of trophectoderm marker *Cdx2* including three germ layer differentiation genes in colonies with *Llgl2* and *Grb7* knockdown (Fig. 4C and Supplementary Fig. 8). Staining the knockdown colonies with AP showed significantly decreased numbers of AP positive ESC colonies compared to controls (Fig. 4D, E). Immunofluorescence staining for OCT4 and SOX2 in knock down ESCs showed loss of expression for both genes when either *Llgl2* or *Grb7* is silenced (Fig. 4F–H). Cell proliferation marker Ki67 is also reduced upon knock down of either gene, while trophectoderm marker CDX2 is increased (Fig. 4I–K).

### Overexpression of *Llgl2* and *Grb7* enhances reprogramming efficiency

**Fig. 5:**
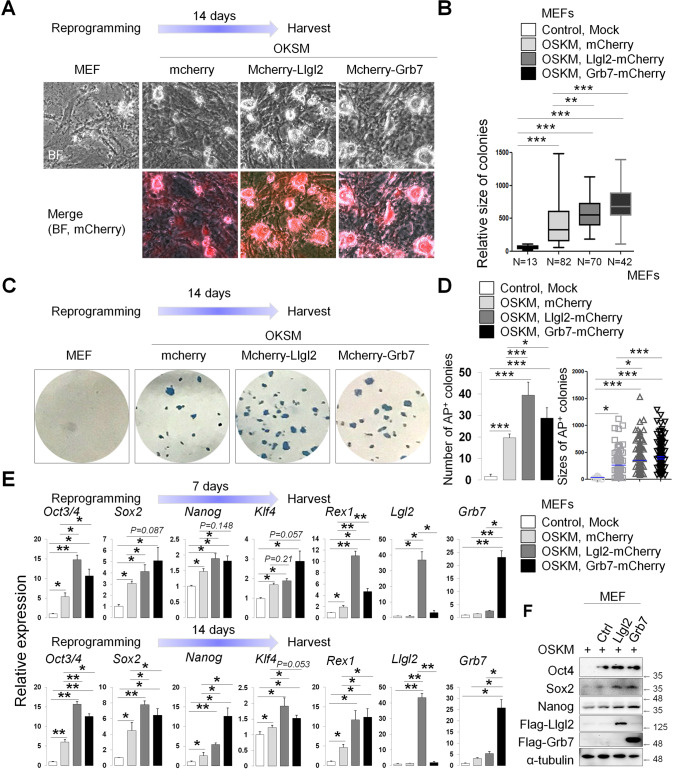
Overexpression of *Llgl2* and *Grb7* enhances reprogramming efficiency. **A** Time course for reprogramming of early passage MEFs with retroviral supernatants containing pMX-hOct4, hSox2, hKlf4, hc-Myc, and either pMX-T2A-mCherry, mLlgl2-T2A-mCherry, or mGrb7-T2A-mCherry. Representative colonies imaged at 14 days post transduction. Images taken at 40X scale; **B** Quantification of reprogramming colony sizes by day 14 post transduction; **C** Alkaline phosphatase staining of colonies on day 14 post transduction. Representative wells shown; **D** Quantification of AP+ colony numbers and size on day 14 post transduction; **E** Quantitative PCR analysis of pluripotency gene expression for reprogramming wells at day 7 and day 14 post transduction; **F** Immunoblotting for pluripotency factors OCT4, SOX2, and NANOG in reprogrammed colonies from MEFs. LLGL2 and GRB7 overexpression confirmed with anti-FLAG antibody. Normalized to α-Tubulin levels. In panels **B**, **D**, **E**, data plotted are mean ± SD for triplicate samples and were compared by one-way ANOVA with Tukey’s post hoc test. (*p < 0.01, **p < 0.001, ***p < 0.0001).

To further elucidate the functional role of *Llgl2* and *Grb7* in pluripotency, we overexpressed these genes in an induced-pluripotent cell (iPS) reprogramming context to determine whether either gene could enhance the establishment of the pluripotent state. We employed a retroviral reprogramming strategy with pMX vectors expressing human *Oct4, Sox2, Klf4* and *c-Myc*. Primary MEFs were additionally transduced with either pMX control, m*Llgl2*-FLAG, or m*Grb7*-FLAG mRNA expressing vectors with mCherry expression to control for transduction efficiency. Wells transduced with either *Llgl2* or *Grb7* showed larger colony sizes compared to controls (Fig. 5A, B). AP staining demonstrated that significantly more AP positive colonies were observed in wells transduced with *Llgl2* and *Grb7* compared to control cells (Fig. 5C, D, left panel). The sizes of these colonies were also larger (Fig. 5D, right panel). Overall, *Llgl2* and *Grb7* could induce more somatic cells to induced-pluripotent stem cells (iPSCs).

Wells were assayed for pluripotency gene expression at 7 and 14 days post transduction. As compared to control transduced colonies, wells overexpressing *Llgl2* and *Grb7* expressed higher levels of pluripotency marker genes *Oct4, Sox2, Nanog, Klf4*, and *Rex1* as soon as 7 days post transduction compared with mock and control wells (Fig. 5E). A similar pattern of expression was observed at 14 days, with expression for *Sox2* and *Nanog* significantly increased in *Llgl2* and *Grb7* overexpressing wells. Comparing protein expressions of OCT4, SOX2, and NANOG, we observe slightly higher expression levels of these pluripotency markers at day 14 (Fig. 5F and Original data file. 1). These experiments demonstrated that *Llgl2* or *Grb7* are required for pluripotency and self-renewal and can enhance reprogramming efficiency to induced pluripotency.

## Discussion

Our study explores how long-range inter-chromosome interactions between the *Oct4* DE and distally located gene loci are directly involved in maintaining the pluripotent state in mESCs. Other studies on enhancer activity and chromatin looping limit their focus on relatively proximal interactions within megabase regions or within the same chromosome, largely due to the lack of resolution and limitations of the assays performed [7, 37, 38]. This potentially overlooks biologically significant long-range interactions that also exert functional influence on transcription programs and cell identity. In the previous study, we observed long-range inter-chromosome interactions between Oct4 locus and candidate gene loci in ESCs. Through analyzing interaction data from our new CRISPR-Dam technique and validating with traditional 3C and DNA-FISH methods, we demonstrate novel interchromosome interaction targets for active super enhancers like the *Oct4* DE that have functional roles in regulating the naïve pluripotent state.

We described a new method to identify these extreme long-range chromosome interactions through utilizing the strengths of the DamID system and CRISPR gene editing. Highly specific targeting of the Nm-gRNA and in vivo labeling of proximal loci by the Dam domain not only allows for mapping of *Oct4* distal enhancer interactome, but also captures interactions at greater resolution than 3C-based methods, which allowed for positive identification of specifically interacting genomic elements in our study. This technique also allows labeling of the transient chromatin interactions. Despite recent reports of endogenous N6-adenine methylation and putative adenine demethylases in mammalian embryonic stem cells, the DamID technique remains viable as the Dam labeling consensus motif is unique and not utilized in endogenous adenine methylation mechanisms [39, 40]. Albeit, our method does have certain shortcomings. Temporal control of Dam activity is a critical parameter as noted in other studies utilizing Dam. Label oversaturation can occur if Dam is allowed to be active for extended periods of time and lead to labeling at unrelated loci. We incorporated the inducible tetracycline expression system to restrict Dam expression and included control cell lines to minimize signal leakage, but more precise control of Dam activity can be achieved such as utilizing ligand-dependent or dimer activated Dam constructs seen in other studies [41, 42]. Further refinement of the protocol to incorporate use of N6-adenine-specific antibodies and employing single cell analysis may also help improve sensitivity and resolution of the assay.

Within the context of the pluripotency gene regulatory network, the focus on the core pluripotency factors, OCT4, SOX2, and NANOG, emphasizes the role of the protein expression in directly mediating pluripotency. OCT4 is recognized as the “master regulator” of the pluripotent state and responsible for activation of other pluripotency-associated genes in somatic cell reprogramming and maintenance of the pluripotent state [43]. However, we demonstrate that the role of the *Oct4* DE is equally important for regulation of stem cell pluripotency. As part of the super enhancer region upstream of *Pou5f1*, the DE contains multiple binding sites for pluripotency factors and architectural chromatin proteins that are known to be required for stem cell pluripotency and self-renewal [12, 30]. In our *Oct4* DE CR4 deletion experiments, we observed that maintaining *Oct4* protein expression does not rescue expression of pluripotency-associated genes like *Llgl2* and *Grb7*, indicating that *Oct4* expression alone is not sufficient to maintain pluripotency. In addition, we show that biallelic or monoallelic deletion of the *Oct4* DE in pluripotent cells results in a disrupted pluripotent state, supporting the important role the *Oct4* DE plays in maintaining the stem cell state. In aligning our CRISPR-Dam peaks with epigenetic marks and whole genome RNA-seq, we are able to reveal active participants in the “proximity-mediated pluripotency network” maintained by the *Oct4* DE. This included many already known pluripotency-related genes as well as previously undiscovered genes. This gives credence to our strategy of simultaneous exploration of chromosome structure and gene expression as a highly useful method for characterizing functional genome contacts stemming from a genomic locus of interest.

As a result of our approach, we identified *Llgl2* and *Grb7* as essential pluripotency-associated genes in pluripotent stem cells, although their exact role will require further study. *Llgl2* is known to associate with Scribble and Discs-large to form the Scribble complex, which plays a role in asymmetric cell division through establishment of apico-basal polarity [44]. In the developing embryo, this axis determination marks the first division of cells where the outer layer forms a polarized epithelium that later develops into trophectoderm and an inner layer that remains apolar that contributes to the inner cell mass at later stages. Overexpression of *Llgl2* in the embryo leads to loss of polarity in epithelial cells and expands the basolateral domain [45]. On the other hand, loss of *Llgl2* has been observed to lead to disorganized cell polarity and excess trophectoderm development in early embryos [46]. This uncontrolled growth of *Llgl2* depleted cells also suggests it plays a role in the Hippo pathway, as dysregulated Scribble complex has been shown to downregulate hippo target genes [47]. *Grb7* has known function as an adaptor protein involved in signal transduction pathways mediating cell migration. The protein contains an SH2 domain that is able to bind to several membrane receptors and can be phosphorylated by focal adhesion kinase (FAK) [48, 49]. *Grb7* has also been shown to be involved in EGF-stimulated mRNA nuclear export in neurons and regulates translation of specific mRNA species through direct binding [50]. Further functional characterization of *Llgl2* and *Grb7* in mESCs can clarify their roles within the respective pathways involved in maintaining the pluripotent cell state.

In summary, our study demonstrates that extremely long-range inter-chromosome interactions occur between the *Oct4* DE and biologically important targets in mESCs to maintain pluripotency. As more studies investigating transcription regulation utilize chromosome conformation assays to map interacting targets of enhancers and other genomic loci, it can be expected that more long-range interactions occurring between loci located on different chromosomes will be identified with biological important functions. Given that long-range looping of the chromatin is an energy costly phenomenon to initiate and maintain, those distal gene targets of these interactions may also be determinative to maintain a certain cell identity, where changes causing reorganization of these long-range interactions only occurs during transitions between different cell states [51]. The CRISPR-Dam method described in our study is well suited for characterizing these often difficult to capture long-range interactions and is a useful tool for exploring the 3D chromatin structure in other cell types.

## Materials and methods

### CRISPR-Dam construction

The Dam domain was cloned from pMQ430 (Addgene # 42216) which contained the *E. coli* DNA adenine methyltransferase gene. The domain was fused to the C-terminus of the catalytically dead, N-terminal FLAG-tagged *Neisseria meningitides* Cas9 protein from M-NMn-VP64 (Addgene #48676) in a vector using the FUW backbone containing a Tetracycline Response Element (TRE) promoter. For reverse tetracycline-controlled transactivator expression and clone selection, pSUPER-hygro was used as a backbone with PGK promoter driving rtTA-IRES-hygro expression. Targeting sequences for the Nm-sgRNA were designed using the CRISPR MultiTargeter webtool (http://www.multicrispr.net/basic_input.html) by specifying parameters for 22-24nt sequence length and 3’PAM sequence “NNNNGMTT”. Highly scored sequences within the Oct4 DE were cloned into a modified pLKO.1-puromycin vector containing an optimized Nm-gRNA loop sequence [34].

### T7 endonuclease I assay

Wild-type E14 cells were transfected with Nm-gRNA and catalytically active NmCas9 plasmid pSimpleII-U6-tracr-U6-BsmBI-NLS-NmCas9-HA-NLS (Addgene # 47868) using Lipofectamine 3000 (Invitrogen, Carlsbad, CA) as suggested by the manufacturer. Cells were incubated for 48 h before genomic DNA was harvested and purified with phenol:chloroform:isoamyl alcohol (VWR, Brooklyn, NY). High fidelity PCR was performed using Q5 Master Mix (NEB, Ipswich, MA) and primers flanking the targeted site within a 600 bp window. Amplicons from wildtype and CRISPR targeted samples were mixed 1:1 and hybridized on a thermocycler before treatment with the T7 Endonuclease I (NEB). Products were run on a 1.5% agarose gel to visualize and quantify CRISPR efficiency using ImageJ.

### Lentiviral particle production and transduction

Nm-dCas9-dam and Nm-gRNA plasmids were made into lentiviral particles using 2nd generation packaging plasmids psPAX and pMD2.G. Plasmids were co-transfected into 293 T cells and incubated for 48 h prior to harvest. Viral media was filtered and ultracentrifuged at 28,000 rpm for 90 min. Viral pellets were resuspended in 200 μl 1× PBS overnight and stored at −80C until use. For transduction, 5 × 10^5^ E14 cells were prepared and 10 μl of lentivirus was added to wells for transduction with 5 μg/ml polybrene. Wells were then centrifuged at 1000 × *g* for 1 h and incubated for 24 h. Clones transduced with Nm-gRNA were selected with 1ug/ml puromycin and manually picked for expansion.

### Cell culture

E14 mouse ES cells were grown in ESC media containing GMEM with 15% FBS, 1% NEAA, 1% Sodium Pyruvate, 1% L-glutamine, 1% PenStrep, 1X B-mercaptoethanol, and 1000U LIF. Cells were passaged using 0.05% Trypsin in PBS solution. Primary mouse MEF cells were derived from E12.5 embryos and cultured in DMEM/10% FBS, 1% PenStrep, and 1% L-glutamine for two passages before use. NmCRISPR-Dam cell lines were generated first through selection of rtTA containing E14 ESC clones using 200 μg/ml Hygromycin B (VWR) and then gRNA selection with 1 μg/ml of puromycin (Santa Cruz Biotechnology, Santa Cruz, CA) for 48 h post transduction. Clones were manually selected and genotyped by PCR to confirm construct integration. NmCas9-Dam labeling performed using 5 × 10^6^ cells and induced with a single pulse of 2 μg/ml doxycycline (Sigma, St. Louis, MO). Cells were harvested at 6-hour increments to quantify FLAG-tagged Cas9-Dam construct expression by western blot with anti-FLAG M2 (Sigma, F1804 1:10 000) and anti-β-ACTIN (1:500, Santa Cruz) antibodies. Total RNA was extracted with Trizol reagent (Invitrogen, Carlsbad, CA) and reverse transcribed using iScript (Bio-Rad, Hercules, CA) for RT-PCR validation of Nm-gRNA expression.

### Library preparation and sequencing

Genomic DNA from samples were extracted and purified using Genomic DNA Mini Kit (Bioland Scientific LLC, Paramount, CA). For qPCR preparation, DNA was digested with DpnII for 24 h and purified by ethanol precipitation before assay. For high throughput sequencing library preparation, DNA was subjected to digestion for 24 h with DpnI and ethanol precipitated before ligating PCR adaptors. DNA was then digested for 24 h with DpnII to exclude amplification of fragments containing non-methylated sites. PCR was performed to enrich fragments flanked by methylated GATC sites. Adaptors were removed via sonication and AluI digestion and cleaned up with AMPure XP beads (Beckman Coulter, Indianapolis, IN). Library fragments were further processed using the NEBNext® Ultra™ DNA Library Prep Kit (NEB) and labeled with dual indexes prior to sequencing. Sequencing was performed on the Illumina HiSeq 4000 using a paired end 151 bp read strategy. Sequencing reads were checked for quality by FastQC before adaptor removal and Barrows Wheeler alignment. PCR duplicates were removed, and the results were summarized through visualization with Integrated Genome Browser.

### Chromosome conformation capture 3C

3C assay was performed following steps previously outlined [52]. Briefly, 1 × 10^8^ E14 cells were trypsinized and crosslinked with 37% formaldehyde for 10 min at room temperature. Crosslinking was quenched with ice cold 2.5 M glycine. Cells pelleted were lysed using Dounce homogenizer. Isolated nuclei were resuspended in 1 ml of 1.2X restriction buffer and distributed into 20 Eppendorf tubes. 1% SDS was added, and the samples were incubated at 65 °C, followed by snap cooling and the addition of 10% Triton X-100. Chromatin was then digested overnight with 400U of HindIII or MluCI. Comparison of purified undigested and digested chromatin aliquots by SYBR Green PCR was performed to check digestion efficiency. Only samples with more than 80% were used for further analysis. Inactivation of the restriction enzyme with 10% SDS and heating at 65 °C was performed before the addition of 7.5 ml of ligation cocktail containing 10% Triton X-100, 10 mg/ml BSA, 100 mM ATP, and 10X ligation buffer containing 500 mM Tris pH 7.5, 100 mM MgCl2, 100 mM DTT. 100U of T4 ligase was then added to each sample tube and incubated overnight at 4 °C and 1 h at room temperature the following day. Samples were de-crosslinked overnight at 65 °C with 30 μg/ml Proteinase K and for 1 h at 37 °C with 30 μg/ml RNAse A. Samples were then purified with 7 ml of phenol-chloroform and precipitated with 3 M sodium acetate and 100% ethanol. Library pellets were resuspended in 10 mM Tris pH 7.5 and stored at −20 °C. BAC control libraries were generated following the same procedure using BAC clones RP23-143F14 representative of the Llgl2 locus, RP23-474J5 for the Grb7 locus, and CH29-91H19 for the Oct4 locus. PCR reactions were performed using APEX 2X HotStart Master Mix on the Biorad Bioengine Peltier Thermocycler. Titration curves were generated using BAC control libraries to prevent signal saturation. Amplicons were run on a 2% agarose gel.

### 3C primer design

Genomic maps of the Oct4 distal enhancer locus, Llgl2 locus, and Grb7 locus were downloaded from the UCSC Genome Browser mm9 for identification of appropriate restriction sites. Primer flanking restriction sites were designed using Primer3 and optimized to have a melting temperature of 60 °C when paired with the bait primer located at the Oct4 DE. Primers were designed to the sense strand and located within 150 bp of the restriction site, for a total fragment length of at least 70 bp and <250 bp after ligation with the bait fragment (Supplementary Table 1).

### DNA fluorescence in situ hybridization

Probes were produced from BAC clones used from 3C assays to visualize the chromatin regions tested. BAC clones were gently extracted and purified using Qiagen Large Construct kit (Qiagen, Germantown, MD). For probe generation, BACs were nick translated with biotin-11-dUTP or dioxigenin-11-dUTP or 90 min at 15 °C and quenched with salmon sperm DNA and mouse Cot-1 DNA. Probes were then precipitated with ethanol and resuspended in deionized formamide and 2X hybridization buffer (2X SSC, dextran sulfate, sodium phosphate monobasic). E14 cells were grown on 1% gelatinized glass slides in preparation. Following a previously described protocol, cells were fixed with 4% formaldehyde and permeabilized with 0.5% Triton X-100 on ice briefly. Then, samples were dehydrated sequentially with ice cold ethanol and rehydrated in 2X SSC. Cells were denatured in 50% formamide/2X SSC (pH 7.2) for 30 min at 80 °C and then quenched in 70% ethanol. Probes were added and incubated for 48 h in a humid chamber. Slides were then washed in 50% formamide/2X SSC at 42 °C and incubated with anti-DIG-fluorescein (Sigma Aldrich cat. 11207741910) for 40 min at 37 °C. After washing with 0.1% Tween 20/4X SSC, slides were then incubated with avidin-rhodamine (Vector Labs, Burlingame, CA) for 40 min at 37 °C. Slides were then washed again in 0.1% Tween 20/2X SSC and counter stained with DAPI before mounting. Images were collected using confocal microscopy (AxioImager with AxioCam HRc color camera; Zeiss, Jena, Germany). Overlapping fluorescein and rhodamine signals were considered colocalized loci. At least 200 nuclei were analyzed for a power of 0.8 at an alpha of 0.05.

### CRISPR knock in distal enhancer LoxP mESC construction

Wild-type E14 cells were transduced with separate lentiviral vectors containing rtTA, TRE- mOct4, and pSin-CreER(T2). Clones were identified through genomic PCR and expanded clonally. Targeting sgRNA were designed using the sgRNA Designer tool (portals.broadinstitute.org/gpp/public/analysis-tools/sgrna-design) to target the CR3 and CR4 flanking sequences located within the Oct DE. Using the SpCas9 system, sgRNAs were cloned into LentiCRISPRv2 (Addgene #52961), and co-transfected with repair donor oligos containing the designated loxP sites and altered PAM sequence using Lipofectamine 3000 (Life Technologies). Clones were picked after 48 h and expanded before genotyping for loxP insertion. Screening PCR was performed after treatment with 0.1 M 4-hydroxytamoxifen for 48 hours. Identified mono- and bi-allelic colonies were stably expanded. For the knockout experiment, cells were treated with 0.1 M 4-hydroxytamoxifen for 48 h and 0.5 μg/ml doxycycline for the appropriate condition. Small molecules were added simultaneously according to condition. Total RNA was extracted using Trizol and reverse transcribed with iScript (Bio-Rad) in preparation for qPCR and next generation RNA sequencing. Immunofluorescence staining was performed using antibodies against OCT4 (Rabbit, SantaCruiz, 1:50), SOX2 (Mouse, Santa Cruz, 1:50), LLGL2 (Mouse, Santa Cruz, 1:50), GRB7 (Mouse, Santa Cruz, 1:50), KI67 (Rabbit, Santa Cruz, Abcam), and CDX2 (Mouse, Santa Cruz, 1:50).

### shRNA knock down and RT-qPCR

Knock down of Llgl2 was performed using GIPZ-based sequences procured from GE Dharmacon. Grb7 KD sequences were cloned into pLKO.1 backbones. Sequences are listed in (Supplementary Table 2). Vectors were transfected using Lipofectamine 3000 with a vector: reagent ratio following manufacturer’s protocol. Transfected E14 cells were incubated for 16 h at 37 °C before selection with 1 μg/ml puromycin for 36 h. Cells were harvested for RNA 72 hours post transfection using Trizol reagent (Life Technologies) and purified by ethanol precipitation. RNA was measured using NanoDrop and normalized to 1 μg for reverse transcription using iScript (Bio-Rad).

### Reprogramming

Llgl2 and Grb7 mRNA were cloned via Gateway Cloning into pMX retroviral plasmid backbone containing a T2A-mCherry in frame. Retroviral particles of the Llgl2 and Grb7 overexpression constructs along with pMX-human Oct4, Sox2, Klf4, and c-Myc (OSKM) were transfected into Plat-E retrovirus packaging cells. Viral supernatants were harvested 3 days post transfection and used freshly. 1 × 10^5^ early passage MEFs were plated in 6-well plates 1 day before transducing twice by spinfection on subsequent days with viral supernatants and 8ug/ml of polybrene. MEFs transduced with OSKM vectors with or without Llgl2 or Grb7 vector were cultured onto gelatin-coated dish with ESC media for 7 or 14 days. iPS colonies emerged by day 14 and entire wells were stained with Alkaline Phosphatase (Vector labs) for quantification. Picked iPS colonies were expanded for 2 days before harvesting mRNA for qPCR quantification and western blot analysis using anti-OCT4 (1:1000, Abcam Ltd, Cambridge, MA), anti-SOX2 (1:1000, Abcam), and anti-NANOG (1:1000, Santa Cruz).

### Real-time quantitative reverse transcription PCR (qRT-PCR)

Cells were harvested and total RNA was isolated using TRIzol reagent (Invitrogen). A SuperScript III qRT-PCR kit (Invitrogen) was used to synthesize cDNA from total RNA. Quantitative PCR was performed using a ViiA7 system (Applied Biosystems, Rockford, IL) or LightCycler 480 system (Roche, Indianapolis, IN) with iTaq Universal SYBR Green Master Mix (BioRad); conditions were 95 °C for 10 min followed by 50 cycles at 95 °C for 15 s and 60 °C for 3 s. Samples were run in triplicate and transcripts were quantitated by comparing Cycle Threshold (Ct) values for each reaction with a *GAPDH* reference. Primer sets for quantitative PCR are listed in Supplementary Table 3.

### Statistical analysis

Statistical analyses were performed using Excel statistical tools or Prism 6 (GraphPad Software). Where differences between treatment groups were experimentally hypothesized, Statistical differences among two groups were analyzed using Student’s *t*-test (*P < 0.05, **P < 0.005, and ***P < 0.0005.). ANOVA tests (Tukey’s multiple comparison test) were used to test hypotheses about effects in multiple groups. Differences are indicated in figures as follows: *P < 0.01, **P < 0.001, and ***P < 0.0001. *P < 0.01 was considered statistically significant.

## Supplementary information

Original Data File

Supplementary Manuscript

## Data Availability

All data are available in the main text or the supplementary materials. RNA sequencing data have been deposited with the NCBI Gene Expression Omnibus (GEO) with the following accession number: GSE122753.

## Materials availability

Reagents generated in this study will be made available on request. A completed Materials Transfer Agreement may be requested if there is potential for commercial application.
