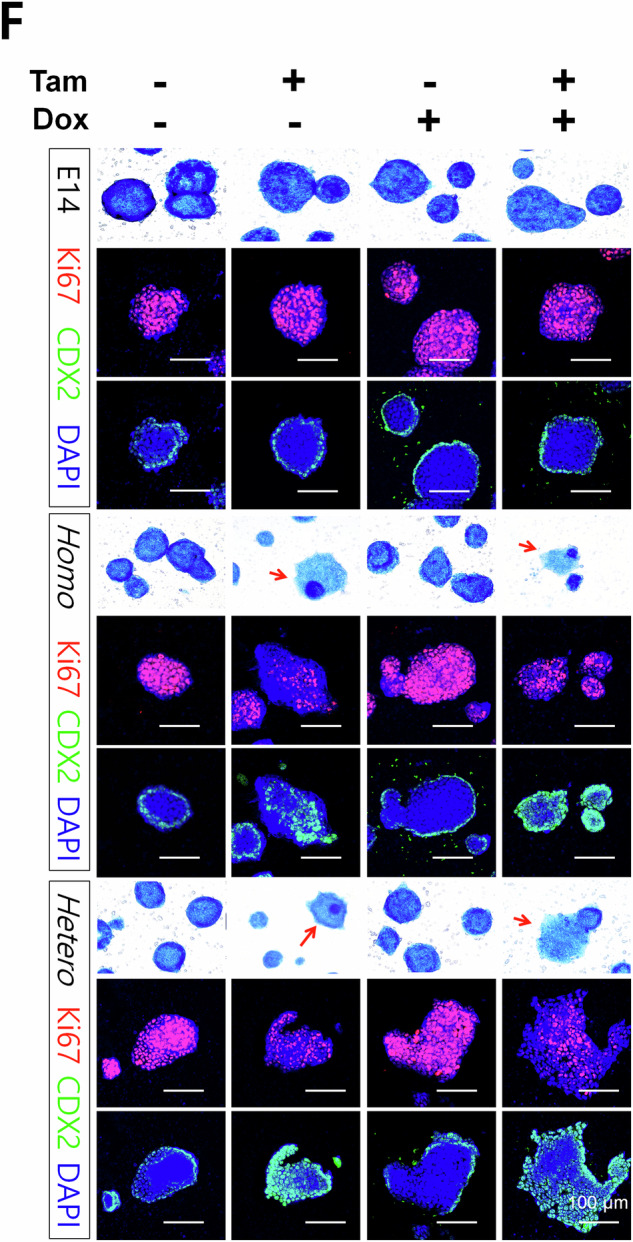# Correction: Long range inter-chromosomal interaction of *Oct4* distal enhancer loci regulates ESCs pluripotency

**DOI:** 10.1038/s41420-024-02125-w

**Published:** 2024-08-20

**Authors:** Byoung-San Moon, David Huang, Fan Gao, Mingyang Cai, Guochang Lyu, Lei Zhang, Jun Chen, Wange Lu

**Affiliations:** 1 Department of Biotechnology, Chonnam National University, Yeosu, 59626 Korea; 2 Department of Stem Cell Biology and Regenerative Medicine, Broad Center for Regenerative Medicine and Stem Cell Research, Keck School of Medicine, University of Southern California, Los Angeles, CA 90033 USA; 3 State Key Laboratory of Medicinal Chemical Biology and College of Life Sciences, Nankai University, 94 Weijin Road, 300071 Tianjin, China

**Keywords:** Nuclear organization, Embryonic stem cells, Nuclear organization

Correction to: *Cell Death Discovery* 10.1038/s41420-023-01363-8, published online 13 February 2023

In this article we have noticed an error on Figure 3F. Specifically, the representative images of merged Ki67-DAPI and CDX2-DAPI for Tam-/Dox+ of the control (E14) and Tam-/Dox- of the homologous (Homo) group were mistakenly duplicated. This unintentional editorial error occurred during alignment of images for figure preparation. We have included a corrected version of Figure 3F. Except for this part, other representative images, including the representative images for alkaline phosphatase staining, and quantification data remain unchanged. There are also no changes to the text and figure legend.

The original article has been corrected.