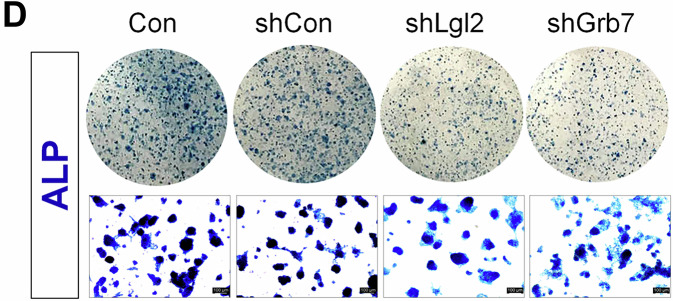# Correction: Long range inter-chromosomal interaction of *Oct4* distal enhancer loci regulates ESCs pluripotency

**DOI:** 10.1038/s41420-026-02993-4

**Published:** 2026-04-14

**Authors:** Byoung-San Moon, David Huang, Fan Gao, Mingyang Cai, Guochang Lyu, Lei Zhang, Jun Chen, Wange Lu

**Affiliations:** 1 Department of Biotechnology, Chonnam National University, Yeosu, 59626 Korea; 2 Department of Stem Cell Biology and Regenerative Medicine, Broad Center for Regenerative Medicine and Stem Cell Research, Keck School of Medicine, University of Southern California, Los Angeles, CA 90033 USA; 3 State Key Laboratory of Medicinal Chemical Biology and College of Life Sciences, Nankai University, 94 Weijin Road, 300071 Tianjin, China

**Keywords:** Nuclear organization, Embryonic stem cells, Nuclear organization

Correction to: *Cell Death Discovery* 10.1038/s41420-023-01363-8, published online 13 February 2023

We have noticed errors in Figure 3F and 4D of our published article. In Figure 3F, the representative merged images of Ki67-DAPI and CDX2-DAPI for Tam-/Dox+ of the control (E14) and Tam-/Dox- of the homologous (Homo) group were mistakenly duplicated during figure preparation. This unintentional error occurred during image alignment. We have included a corrected version of Figure 3F. All other representative images in this panel, including those for alkaline phosphatase staining and the associated quantification data, remain unchanged. In Figure 4D, an inadvertent partial duplication occurred between the first and second upper panels during figure assembly using Adobe InDesign. The Control and shControl wells were correctly stained and imaged in paired upper and lower wells; however, an unintended misplacement during figure assembly resulted in duplication of the shControl image in the panel labeled Control. The four high-magnification representative images shown in the lower panels accurately reflect the original data. Corrected versions of Figures 3F and 4D are provided. These corrections do not affect the text, figure legends, or the conclusions of the study. We sincerely apologize for the oversight and any inconvenience caused to the readers.

Incorrect figure 3F
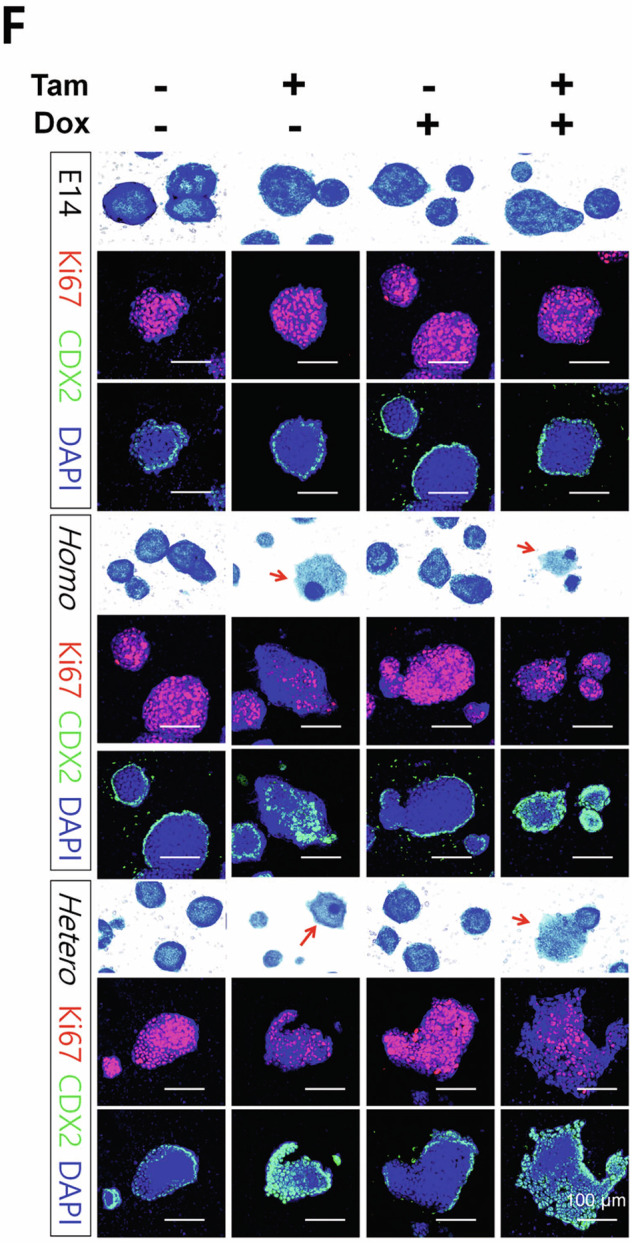


Correct figure 3F
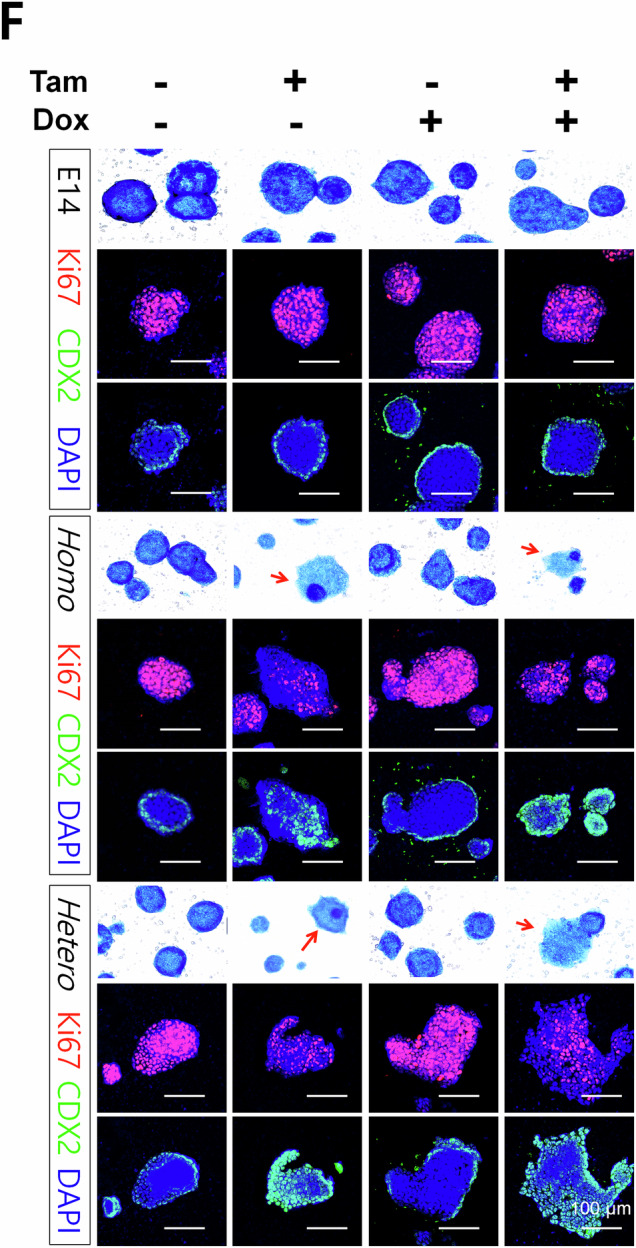


Incorrect figure 4D
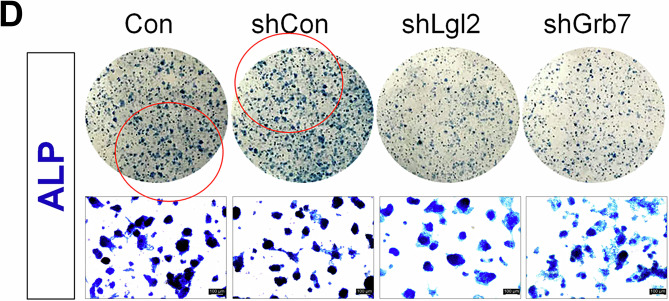


Correct figure 4D