# Supplementary material for: Long range inter-chromosomal interaction of Oct4 distal enhancer loci regulates ESCs pluripotency
Source: Cell Death Discov. 2023 Feb 13;9:61. doi: 10.1038/s41420-023-01363-8 (PMC9925822; doi:10.1038/s41420-023-01363-8)
Supplement: Supplementary file 1 — Original Data File [file 41420_2023_1363_MOESM1_ESM.docx]

**Original data files**

S1

**
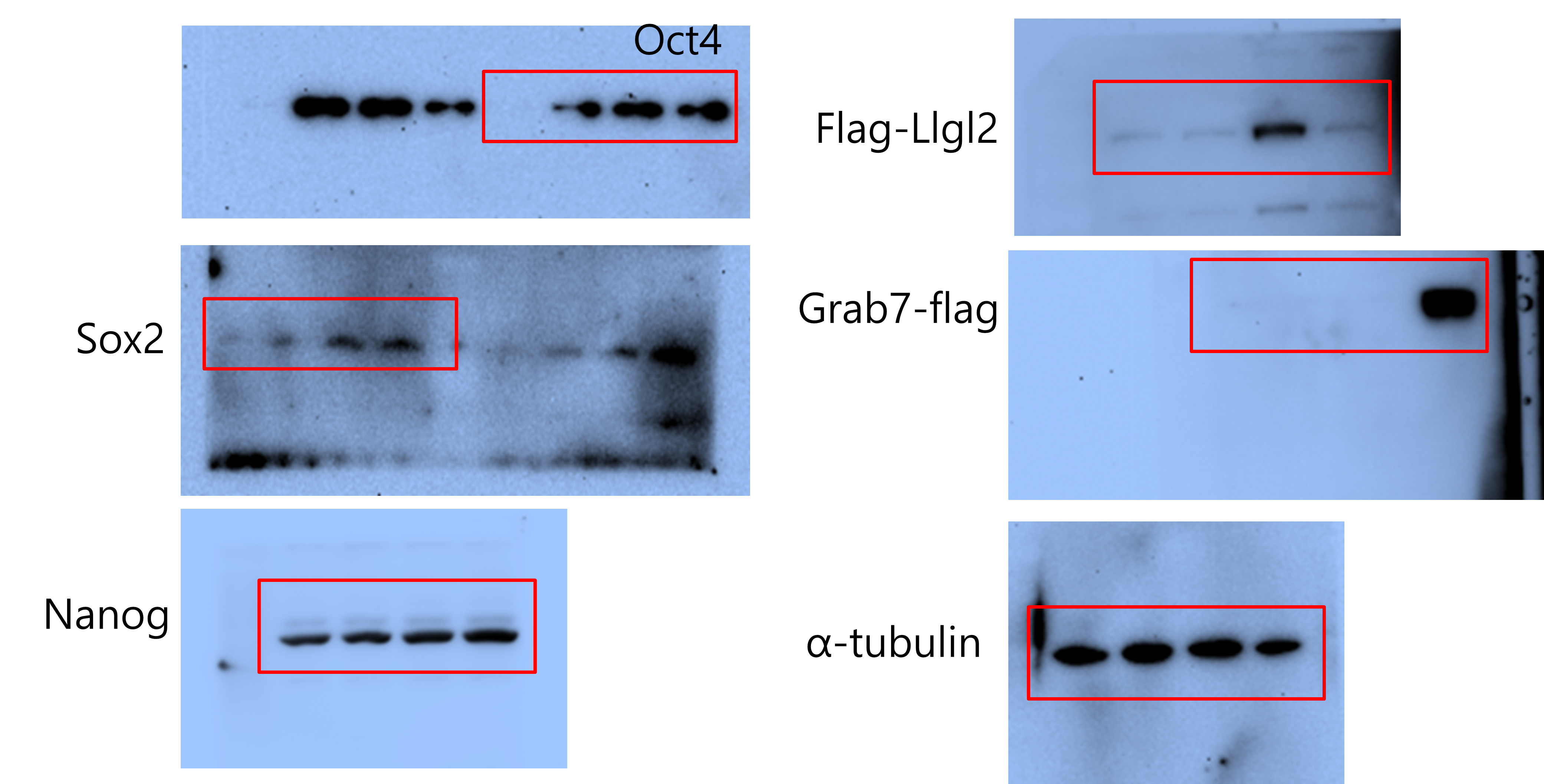
**

**Original data file. 1 Full blots used in main figures.** The full blots used for **Fig. 5F**. The specific bands shown in the main figure are indicated by red boxes. We ran the gel with same samples and each blots were obtained contemporary same period time.

**
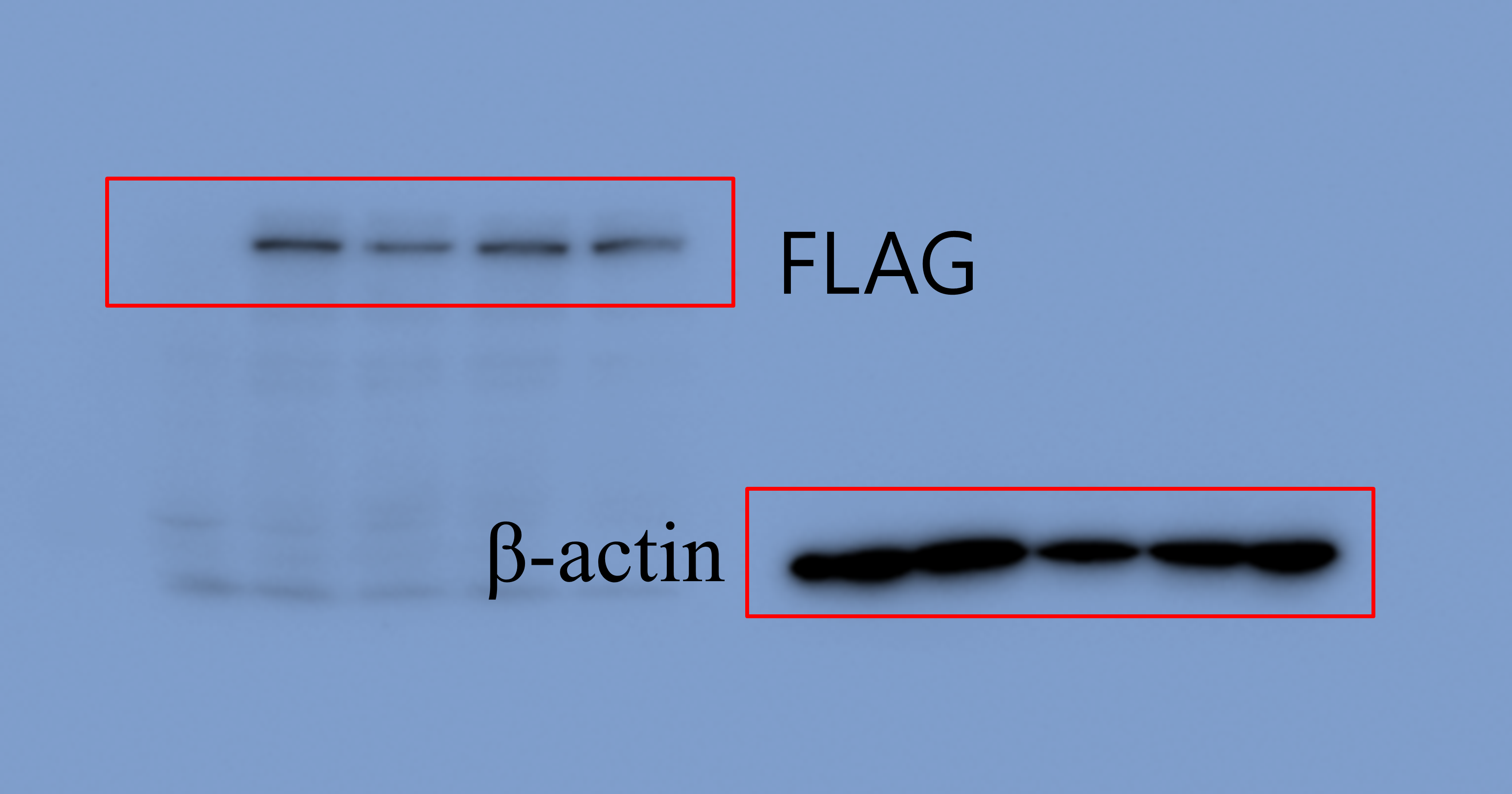
**

**Original data file. 2 Full blots used in main figures.** The full blots used for **Supplementary Fig. S1B**. The specific bands shown in the main figure are indicated by red boxes. We ran the gel with same samples and each blots were obtained contemporary same period time.
