# Supplementary material for: Long range inter-chromosomal interaction of Oct4 distal enhancer loci regulates ESCs pluripotency
Source: Cell Death Discov. 2023 Feb 13;9:61. doi: 10.1038/s41420-023-01363-8 (PMC9925822; doi:10.1038/s41420-023-01363-8)
Supplement: Supplementary file 2 — Supplementary Manuscript [file 41420_2023_1363_MOESM2_ESM.docx]

**Supplementary Figure**

S1


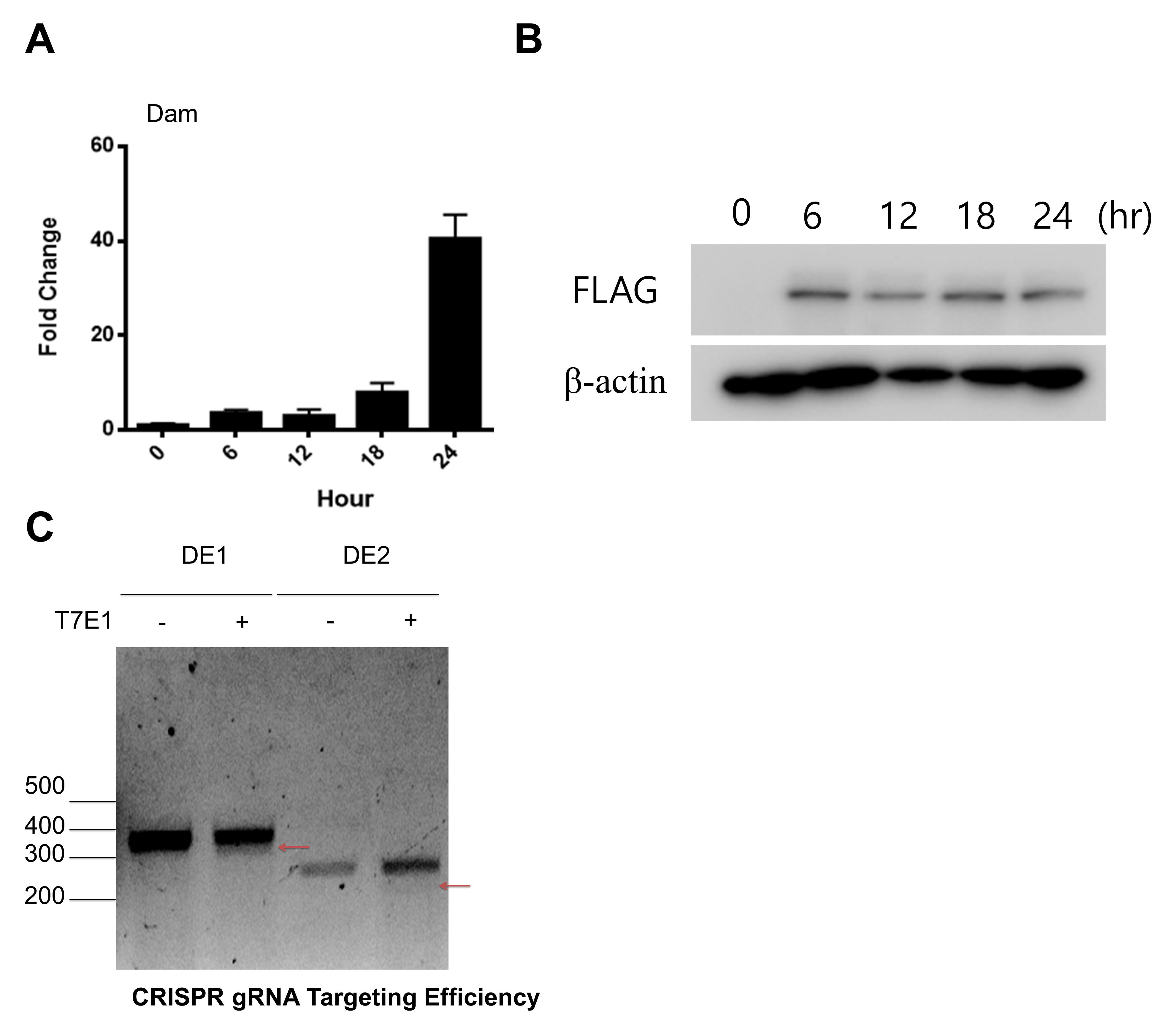


**Fig. 1 Confirmation of CRISPR-Dam assay components.** **A** Dam expression measured by qPCR over 24 hours in 6-hour increments. Signals normalized to β-actin and 0-hour sample. Error bars represent standard deviation in three replicates; **B** Western blot for FLAG-tagged CRISPR-Dam protein expression over 24 hours in 6 hour increments. Signals normalized to β-actin; **C** T7 Endonuclease I assay confirms targeting of two gRNAs targeting the *Oct4* DE locus. (-) T7E1 untreated lanes (+) T7E1 treated lanes. Arrows indicate digested fragments from mismatched CRISPR modified templates.


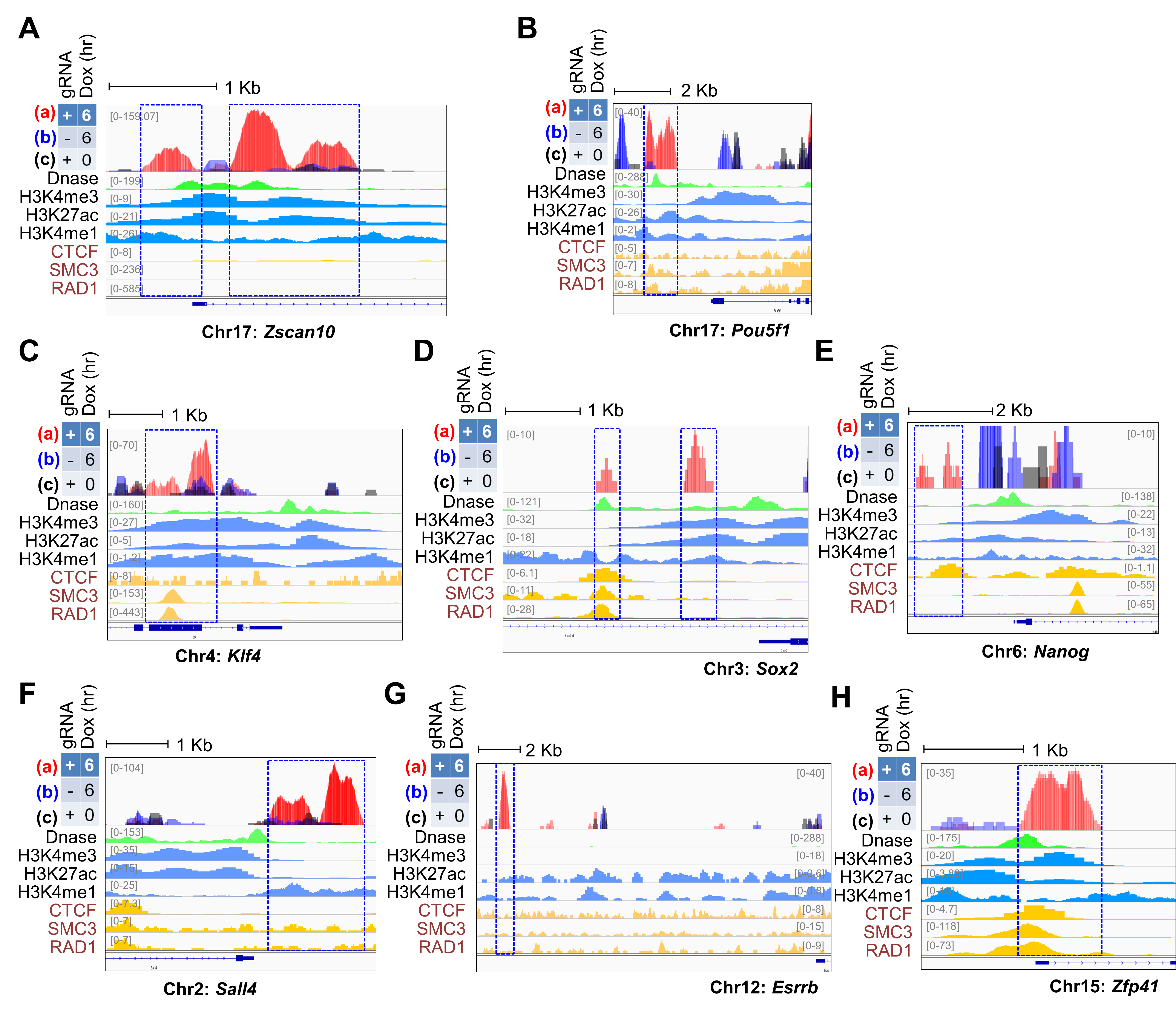


**Fig. 2 CRISPR-Dam sequencing peaks at pluripotency related genes.** **A-H** CRISPR-Dam interaction peaks at pluripotency related genes *Zscan10, Pou5f1, Klf4, Sox2, Nanog, Sall4, Esrrb*, and *Zfp41* with alignment to ENCODE histone modification tracks H3K27ac, H3K4me1, and H3K4me3, DNA accessibility represented by DNaseI hypersensitivity sites, and known architectural proteins CTCF and cohesin subunits SMC3 and RAD21 binding sites. Blue boxed regions indicate CRISPR-Dam peaks regions of interest. Blue, red, and green highlighted regions that are peaks in respective ENCODE tracks. Putative looped out regions are indicated corresponding to architectural protein binding sites.


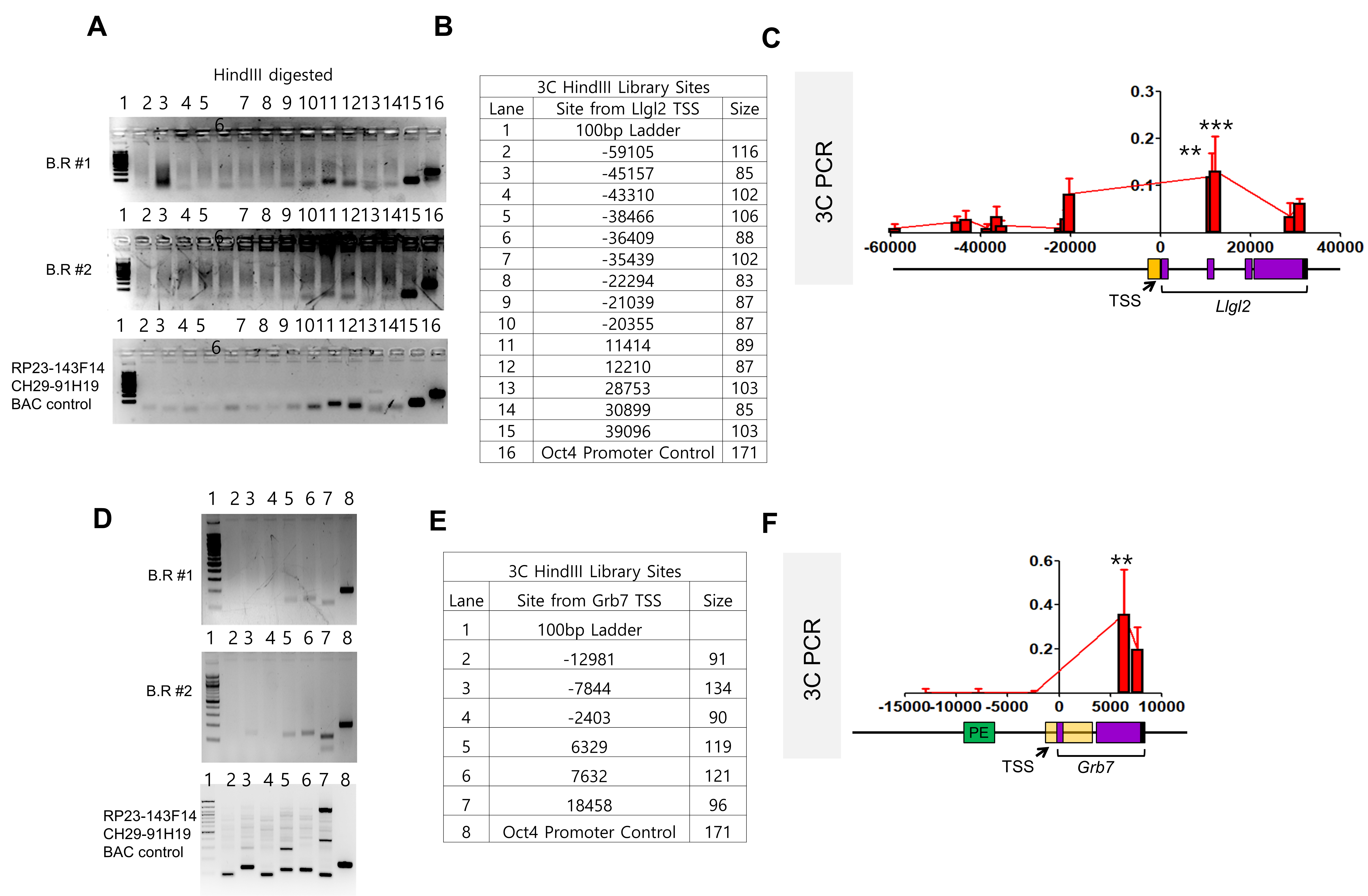


**Fig. 3 Chromosome Conformation Capture assay of *Oct4* DE and *Llgl2* or *Grb7* loci interaction. A** and **B** Chromosome Conformation Capture (3C)-PCR gels from triplicate 3C libraries 1-3. Lanes indicated correspond to HindIII sites at set distances from the TSS of *Llgl2*. Band intensities were measured using ImageJ and normalized to intensities from internal *Oct4* promoter control site and BAC control library; **C** 3C-PCR plots for interchromosomal interaction between the *Oct4* DE locus on chromosome 17 and *Llgl2* regions on chromosome 11. HindIII sites indicated by green arrows. Frequencies calculated from 3C-PCR band intensities averaged over triplicate samples and normalized to an internal control. Error bars indicate standard deviation for biological triplicates; **D** and **E** 3C-PCR gels from triplicate 3C libraries 1-3. Lanes indicated correspond to HindIII sites at set distances from the TSS of *Grb7*. Band intensities were measured using ImageJ and normalized to intensities from internal Oct4 promoter control site BAC control library. **F** 3C PCR plots for interchromosomal interaction frequency between the *Oct4* DE locus on chromosome 17 and *Grb7* regions on chromosome 11. HindIII sites indicated by green arrows. Frequencies calculated from 3C-PCR band intensities averaged over triplicate samples and normalized to an internal control. Error bars indicate standard deviation for biological triplicates.


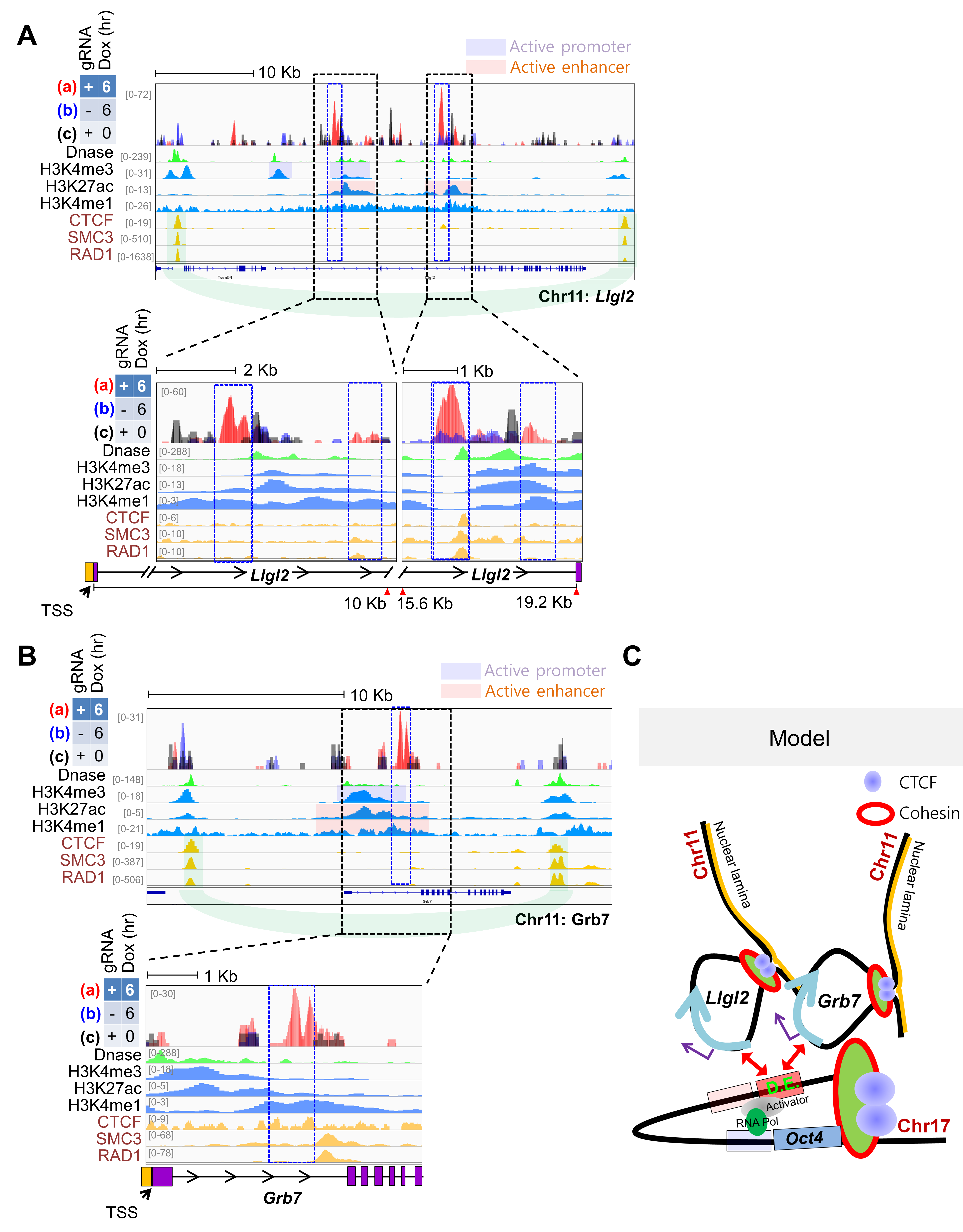


**Fig. 4 The *Oct4* Distal Enhancer Locus Interacts with Promoter Regions of *Llgl2* and *Grb7*. A** and **B** Profile of CRISPR-Dam peaks at *Llgl2* and *Grb7* loci in chromosome 11. ENCODE tracks are included for histone modifications H3K27ac, H3K4me1, and H3K4me3, DNA accessibility represented by DNaseI hypersensitivity peaks, and architectural protein binding sites for CTCF, SMC3, and RAD21. Boxed regions (in blue) highlight CRISPR-Dam peaks aligned with active histone marks. Distinct regions flanked by architectural proteins binding sites suggest these regions may be looped out (red and green highlight); **C** A model indicates *Lgl2* and *Grb7* interact with *Oct DE* through a form of chromatin looping by CTCF and cohesion components such SMC3 and RAD21.


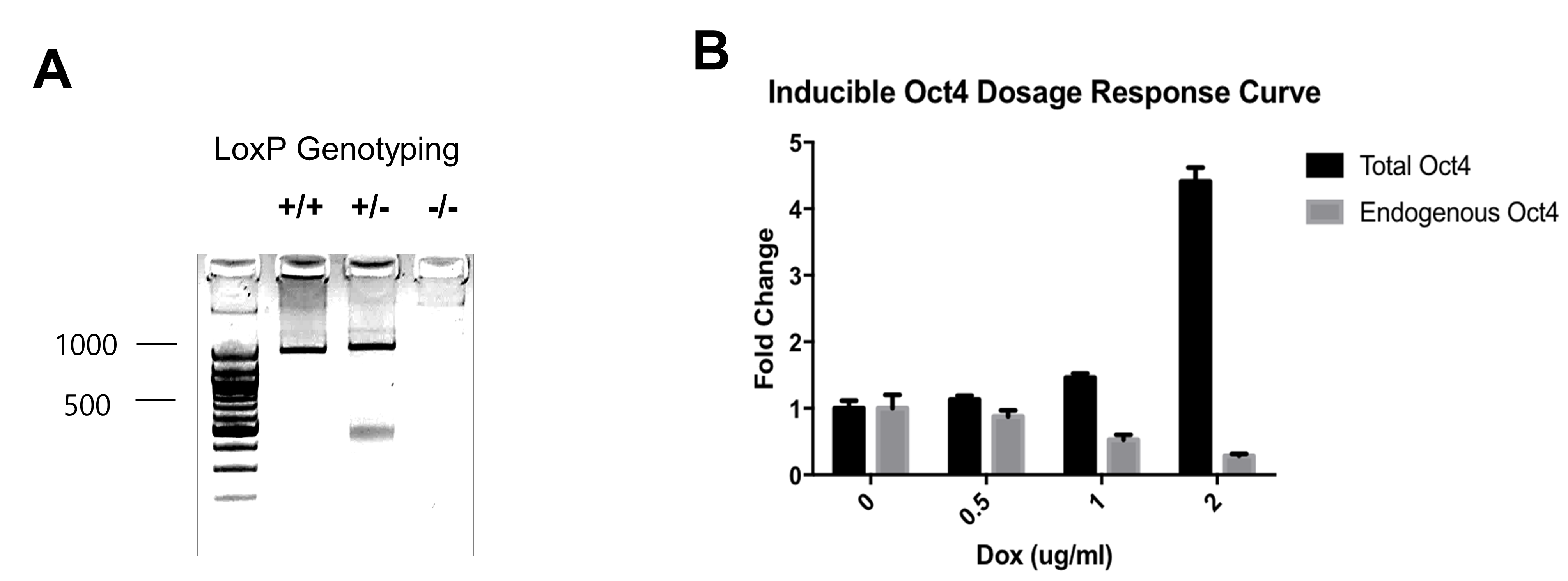


**Fig. 5** ***Oct4* DE deletion construct validation. A** PCR genotyping of CR4 locus in wildtype (+/+), heterozygous (+/-), and homozygous (-/-) loxP inserted E14 cell lines after treatment with 0.1M tamoxifen for 48 hours; **B** Induction of transgenic *Oct4* by increasing doxycycline doses in CR4 loxP inserted E14 cell lines. Cells were treated with doxycycline for 48 hours before harvest and qPCR. Total Oct4 and endogenous Oct4 levels were measured to confirm transgenic Oct4 activation. Signals were normalized to β-actin.


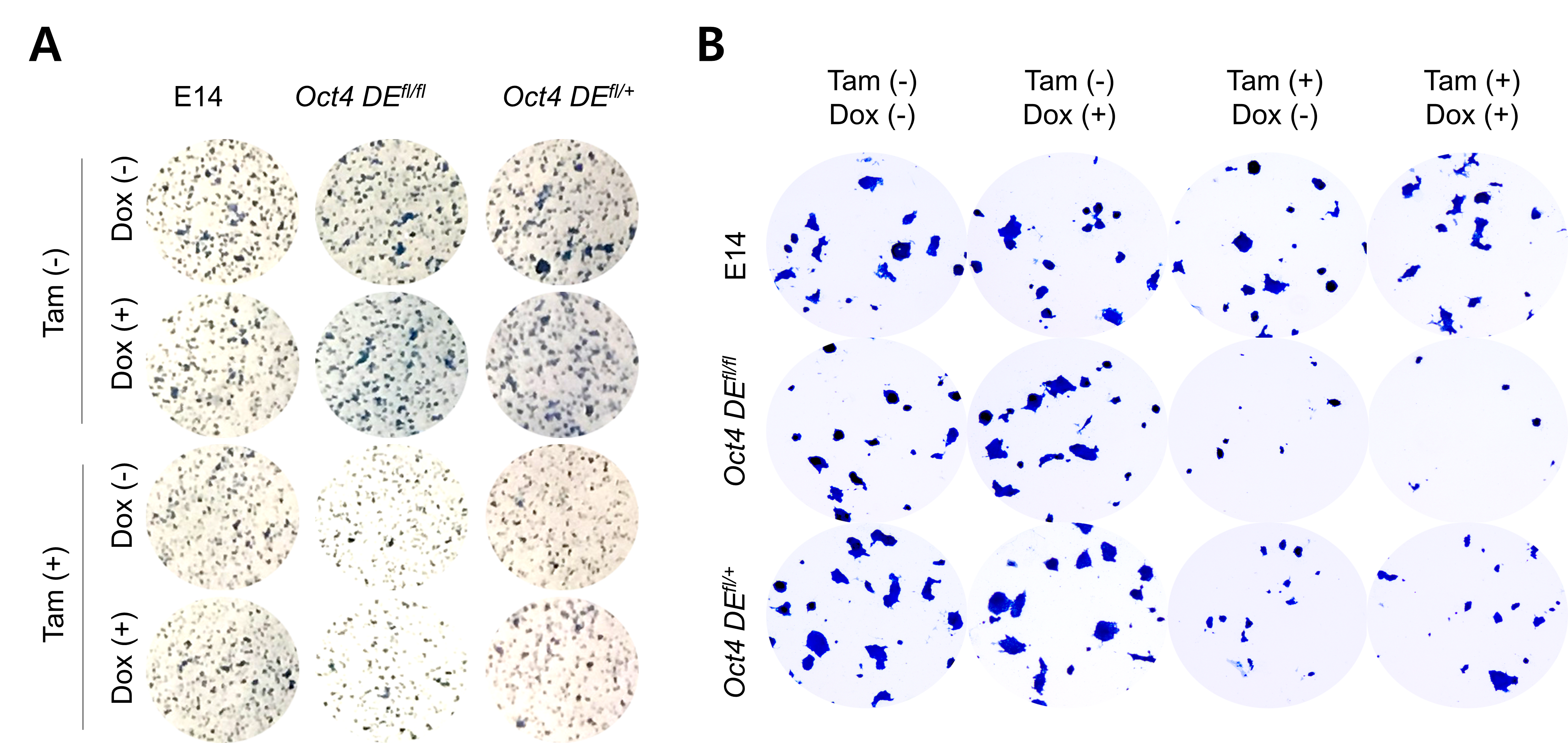


**Fig. 6 AP staining of *Oct4* DE deletion ESCs.** **A** Comparison of wildtype E14 and Oct4 DE homozygous (-/-) and heterozygous (+/-) deletion cell lines with addition of tamoxifen (Tam) or doxycycline (Dox) conditions. 10X magnification; **B** Higher magnification of AP-stained ESC colonies in Tam or Dox conditions. 40X magnification.


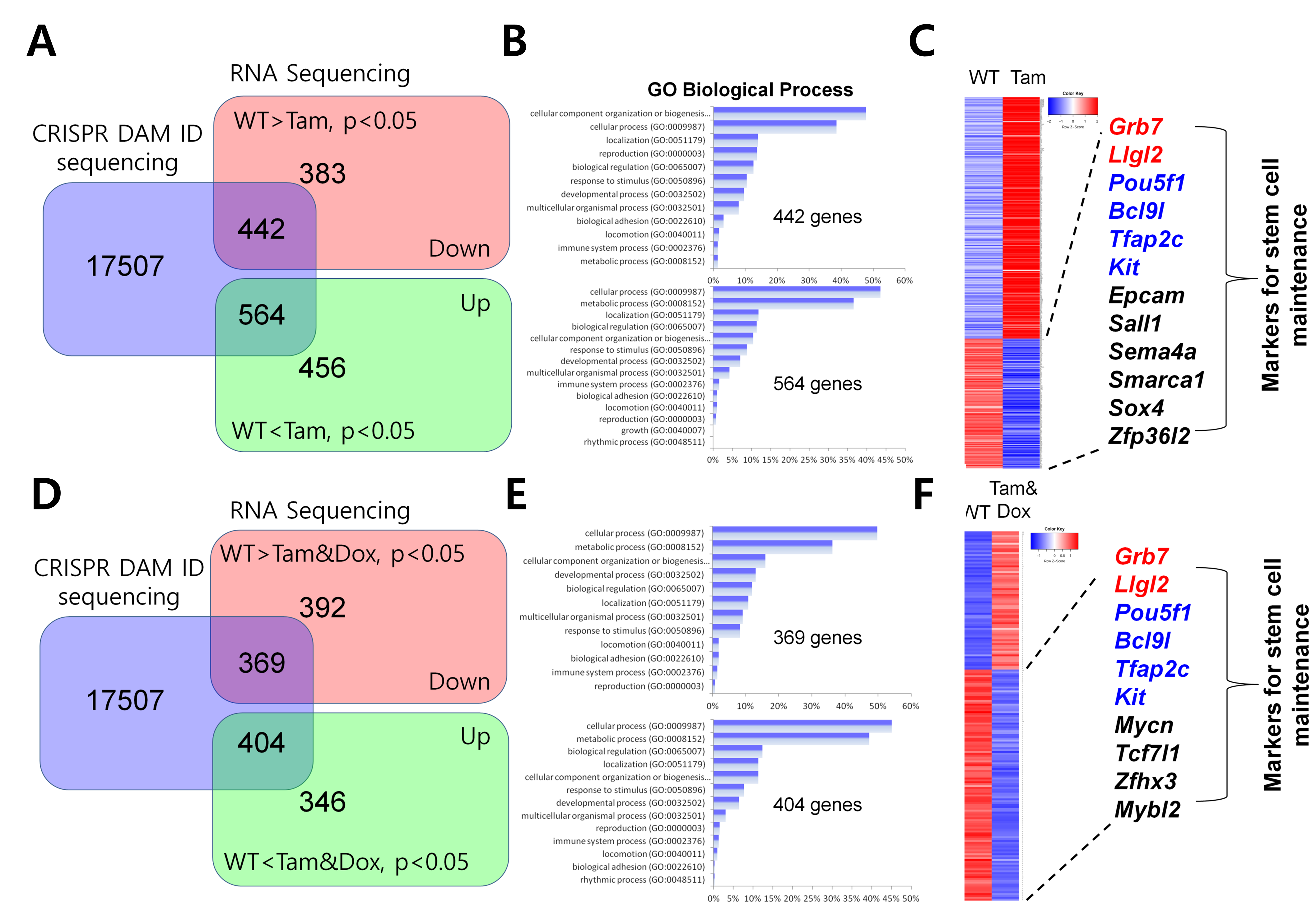


**Fig. 7 CRISPR-Dam peaks overlap with RNA-seq of Oct4 DE floxed ESCs identifies Oct4 DE regulated genes.** **A** Overlap of CRISPR-Dam identified peaks with significantly changing genes (p<0.05) identified in RNA-seq of global gene expression in Oct4 DE (-/-) cells treated with tamoxifen; **B** Unique overlapping genes were analyzed for gene ontology as indicated; **C** Heatmap showing comparison of differentially expressed CRISPR-Dam identified genes in wildtype and Oct4 deleted cells. Down regulated genes in Tam condition comprise of genes involved in stem cell maintenance; **D** Overlap of CRISPR-Dam identified peaks with significantly changing genes (p<0.05) identified in RNA-seq of global gene expression in Oct4 DE (-/-) cells treated with tamoxifen and doxycycline; **E** Unique overlapping genes were analyzed for gene ontology as indicated; **F** Heatmap showing comparison of differentially expressed CRISPR-Dam identified genes in wildtype and Oct4 deleted cells. Down regulated genes in Tam and Dox condition comprise of genes involved in stem cell maintenance.


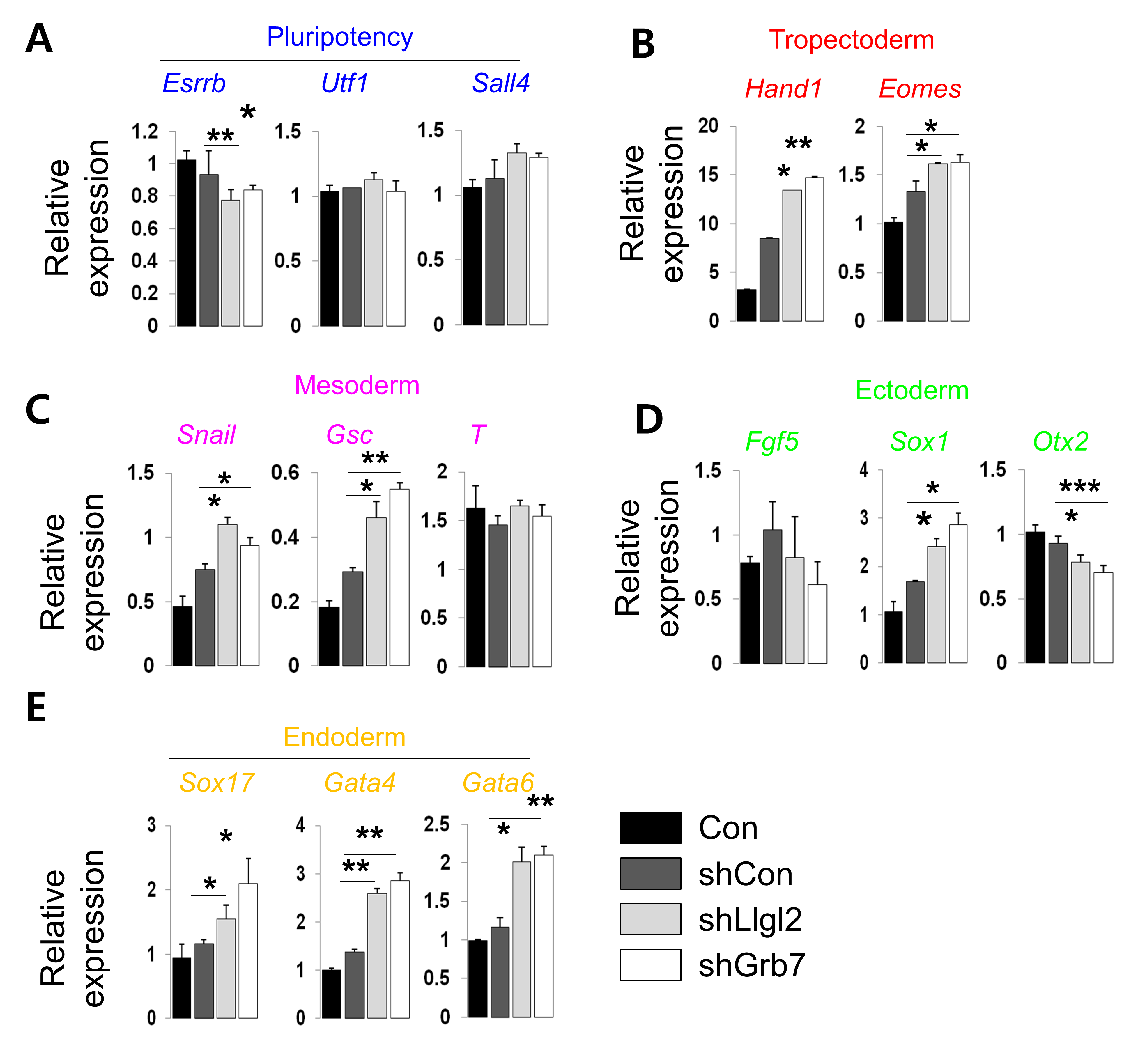


**Fig. 8 Knock down of *Llgl2* and *Grb7* Abrogates mESC Pluripotency.** **A** qPCR for pluripotency genes (*Esrrb, Utf1, Sall4*) and differentiation genes for trophectoderm (*Cdx2, Hand1, Eomes*), ectoderm (*Fgf5, Sox1, Otx2*), mesoderm (*Snail, Gsc, T*), and endoderm (*Sox17, Gata4, Gata6*) in shRNA pool knockdown ESCs. (**p*<0.01, ***p*<0.001, ****p*<0.0001)

**Supplementary Table**

S1

**Supplementary Table 1. 3C PCR primers**

| **Distance to Llgl2 TSS** | **Sequence** | **Fragment Size** |
| --- | --- | --- |
| -59105 | GGGTTCCCAAGCGATTTCTC | 116 |
| -45157 | GTAACCCAGGGCTCTCAGAA | 85 |
| -43310 | CGTGACTGGAAAGAGGCTGA | 102 |
| -38466 | ACACCTCAAAGAACGGAAGAA | 106 |
| -36409 | GGAGTCCTTAGGCTAGATTTTGA | 88 |
| -35439 | TGACTAGGGAATGGCAGGAC | 102 |
| -22294 | CCAGGGAGCGGAGAACAA | 83 |
| -21039 | GCTGACGTCTATTTCCTGTCG | 87 |
| -20355 | GCAGGTTGTGATATCCGGGA | 87 |
| 11414 | CCCTGATACTGTCCCCAAGG | 89 |
| 12210 | ATGACACCTGCCCCTGGA | 87 |
| 28753 | GCATGTCCATTTTAGAGGAGGG | 103 |
| 30899 | TGTCTTGGGGTACAGAGCAA | 85 |
| 39096 | CCAAGCCAGTTTCTCTACCC | 103 |
| **Distance to Grb7 TSS** | **Sequence** | **Fragment Size** |
| -12981 | TCCTGTCCTTTCATGGGTTC | 91 |
| -7844 | CCAAGTGAGGGGCTAAAGGT | 134 |
| -2403 | CAAAGCCATGGAACAACAAA | 90 |
| 6329 | CTGAGATCCAGGGCTTCCT | 119 |
| 7632 | GCAGCTGAGCACAGAATCAT | 121 |
| 18458 | GTTGAGGGCTCCTTCCTACC | 96 |
| InternalControl_F | CCCTCCTCCTAATCCCGTCT | 171 |
| InternalControl_R | CCCCTCACACAAGACTTCCC |  |
| DE Bait for HindIII | GGCAGAGAAGATGGTTGGGG |  |

**Supplementary Table 2. 3C PCR densitometry analysis**

**Supplementary Table 3. CRISPR-DAM ID qPCR primers**

| **Llgl2 Loci GATC sites** | **F/R** | **Primer Sequences** | **Size** |
| --- | --- | --- | --- |
| **L11** | **Forward** | **AGTCCCTGAAAGTGGGACCT** | **154** |
|  | **Reverse** | **AAAGGTGGGTAAGACCGTGA** |  |
| **L12** | **Forward** | **GAGGCCATGTGTTCACCTTT** | **164** |
|  | **Reverse** | **AGGGAGCCTTGGGTGTTAGT** |  |
| **L13** | **Forward** | **CCCAGAGGAGAAGTCTCAGG** | **155** |
|  | **Reverse** | **GGGTTCCCTTAGCTCCATTC** |  |
| **L14** | **Forward** | **GGGGTGTGACCGAGAGAGT** | **189** |
|  | **Reverse** | **CACCAGGACTCAGCATCTGT** |  |
| **L15** | **Forward** | **GTAGAGACCGGGAACAGCAA** | **191** |
|  | **Reverse** | **TTTATCAGGGTCGAGCAAGG** |  |
| **L16** | **Forward** | **TGTAGCCACAGACGCAGAAC** | **276** |
|  | **Reverse** | **CATCAGCAAGGTGTGTCGTC** |  |
| **L17** | **Forward** | **GGTGCAGAAGGGTTAAGTGC** | **140** |
|  | **Reverse** | **CAAGCCCAGGACTTAGGTGA** |  |
| **L18** | **Forward** | **CTTTTGCCTTACAGCCTTGC** | **146** |
|  | **Reverse** | **GGAACATCCCCACTCTGGTA** |  |
| **L19** | **Forward** | **CCCTCCCTTCATTCCCGATT** | **179** |
|  | **Reverse** | **CTACCACCACCCAGCTGTT** |  |
| **L20** | **Forward** | **CAGCGGCCCCTCTGAAGA** | **236** |
|  | **Reverse** | **GCACAAGAGCCAAGCGAG** |  |
| **Grb7 Loci GATC sites** | **F/R** | **Primer Sequences** | **Size** |
| **G21** | **Reverse** | **TATTCTCCCGCCTCAACATT** | **153** |
|  | **Forward** | **GAGCACCTCCAACAGAAGGA** |  |
| **G23** | **Reverse** | **TCCTTCCCCTTCCTCATCTT** | **246** |
|  | **Forward** | **GGACCACAACGTGGAGAGAT** |  |
| **G24** | **Reverse** | **GGGAGTAAGGGAGAGCGACT** | **151** |
|  | **Forward** | **CACACTACACCCACCCCTCT** |  |
| **G26** | **Reverse** | **CCAACGGAACCTTTGCTTAG** | **181** |
|  | **Forward** | **AAGCTCAGGTGCAGGCTAAA** |  |
| **G28** | **Reverse** | **AGAGGCAGGTTGTTTGTGCT** | **188** |
|  | **Forward** | **GACTGTGAGGCTACCCAGGA** |  |
| **G29** | **Reverse** | **CTGGGATTGGCATTTTGTCT** | **189** |
|  | **Forward** | **CTGAAAGCACCTGGCATACA** |  |
| **G30** | **Reverse** | **AAAGGACGCACATCCTGTTC** | **120** |
|  | **Forward** | **ATAGAGAAAGGGCGGAGCTG** |  |
| **G31** | **Reverse** | **CAGCTCCGCCCTTTCTCTAT** | **211** |
|  | **Forward** | **CCTACCAATGCATTCCCATT** |  |
| **GAPDH control** | **Forward** | **GGGAGAACAGGGGAAATGGA** | **176** |
|  | **Reverse** | **CCACCCTGGCATTTTCTTCC** |  |

**Supplementary Table 4. DNA FISH Probes and Colocalization Results**

| Probe Target | BAC Name | Region Aligned in mm9 |
| --- | --- | --- |
| Llgl2 | RP23-143F14 | chr11: 115559980-115767566 |
| Grb7 | RP23-474J5 | chr11: 98210673-98380153 |
| Thy1 | CH29-562H16 | chr9: 43679498-43876156 |
| Oct4  Col1a1 | CH29-91H19  RP23-75B6 | chr17: 35417731-35706656  chr11:94748953-94970343 |
|  |  |  |


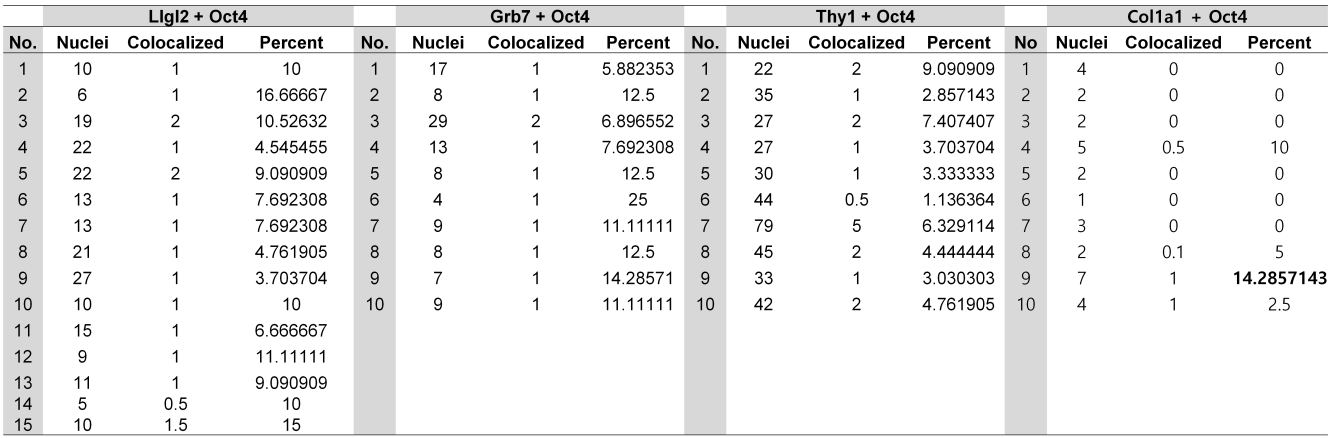


**Supplementary Table 5. CRISPR Knock-In LoxP**

|  |  |  |
| --- | --- | --- |
| **sgRNA** |  |  |
| CR4-sgRNA_F | CACCGCCCACACCCAGTTCCTCCCA | |
| CR4-sgRNA_R | AAACTGGGAGGAACTGGGTGTGGGC | |
| DE3'-sgRNA_F | CACCGCAGTCTGAGGATCCCATTAC | |
| DE3'-sgRNA_R | AAACGTAATGGGATCCTCAGACTGC | |
|  |  |  |
| **Repair Template Oligos** |  |  |
| CR4-donor | GGCTGCAGGCATACTTGAACTGTGGTGGAGAGTGCTGTCTAGGCCTTAGAGGCTGGTCCTGGGAGGAACTGGGTGTGGGGAGGATAACTTCGTATAGCATACATTATACGAAGTTATTTGTAGCCCGACCCTGCCCCTCCCCCCAGGGAGGTTGAGAGTTCTGGGCAGACGGCAGATGCATAACAAAGGTGCATGATAGC | |
| DE3'-donor | TCCCTTGCAGACAGGCACTCTGAGGGCTATTCTCTTGCAAAGATAACTAAGCACCAGGGCAGTAATGATAACTTCGTATAGCATACATTATACGAAGTTATGGATCCTCAGACTGGGCCCAGAAAACCACTCTAGGGAAGTTCAGGGTAGGCTCTCTGCACCCCCTCCTCCTAATCCCGTCTCCTTAGTGTCTTTCCGCC | |
|  |  |  |
| **LoxP Genotyping** |  |  |
| CR4flankF1 | GTCTGCTGTCCCATCTCCAG | |
| CR4flankR1 | GGACTCCGGTGTTCATCCTC | |
|  |  |  |
| **Cre Genotyping** |  |  |
| CreGenoF | GAACCTGATGGACATGTTCAGG | |
| CreGenoR | AGTGCGTTCGAACGCTAGAGCCTGT | |

**Supplementary Table 6. Target sequences of shRNA**

| **Llgl2 shRNA Sequences** | |  |
| --- | --- | --- |
| No. | GE Dharmacon No. | Targeted Sequence |
| 1 | 25726 | TATCAGATAGAAACCTTGG |
| 2 | 31673 | TTCGTTATAGCTTTGCAAG |
| 3 | 459624 | CTGCATCTCAGTGGTCCAC |
| **Grb7 shRNA Sequences** | |  |
| No. | TRC No. | Targeted Sequence |
| 1 | TRCN0000097204 | CTCTCTCAACCAGTGAAACAT |
| 2 | TRCN0000097207 | CTTCGCCAAGTATGAACTATT |
| **Scramble shRNA** | |  |
| No. | N/A | Targeted Sequence |
| 1 |  | GTTAACTATCCTAGCTCTAA |

**Supplementary Table 7. Genotyping results with the Sanger sequencing.**

| **A. Oct4 DE 3’loxP** |
| --- |
| TCTGTCCTGGCTATGTACACTGTGGGGTGCTCTGGGCTTTTTGAGGCTGTGTGATTCACCCTGGGGCCTTCGTTCAGAGCATGGTGTAGGAGCAGACAGACAAACACCATCCCTTGCAGACAGGCACTCTGAGGGCTATTCTCTTGCAAAGATAACTAAGCACCAGGGCAGTAATGATAACTTCGTATAGCATACATTTACGAAGTTATGGATCCTCAGACTGGGCCCAGAAAACCACTCTAGGGAAGTTCAGGGTAGGCTCTCTGCACCCCCTC |
| 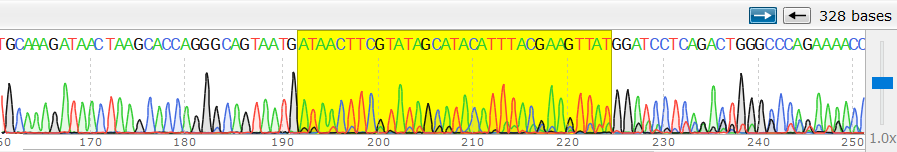 |
|  |
| **B. Oct4 DE CR4-5’loxP** |
| GACAGGACTGCTGGGCTGCAGGCATACTTGAACTGTGGTGGAGAGTGCTGTCTAGGCCTTAGAGGCTGGCCCTGGGAGGAACTGGGTGTGGGGAGGATAACTTCGTATAGCATACATTTACGAAGTTATTTGTACCCCGACCCTGCCCCTCCCCCCAGGGAGGTTGAGAGTTCTGGGCACACGGCAGATGCCTAACAAAGGTGCATGATAGCTCTGCCCTGGGGGCAGA |
| 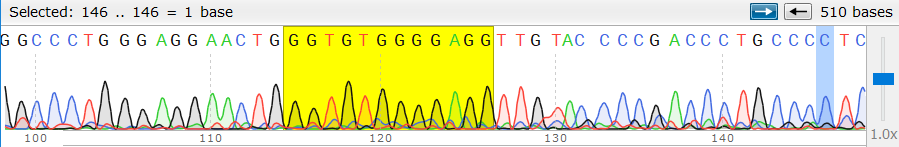 |

Red = loxP site

**Supplementary Table 8. Primer sequences used for quantitative RT- PCR analysis.**

| **Gene** | **F/R** | **Primer Sequences** | **Tm (℃)** |
| --- | --- | --- | --- |
| *mOct3/4*  *(total Oct4)* | Forward | GGCGTTCTCTTTGGAAAGGTGTTC | 68 |
|  | Reverse | CTCGAACCACATCCTTCTCT |  |
| *mOct3/4*  *(endogenous Oct4)* | Forward | CCTGGCCTGTCTGTCACTCA | 69 |
|  | Reverse | GTGTCCCAGTCTTTATTTAAGAACAAAAT | 63 |
| *mNanog* | Forward | CTCTTCAAGGCAGCCCTGAT | 67 |
|  | Reverse | CCATTGCTAGTCTTCAACCAC | 63 |
| *mSox2* | Forward | GGTTACCTCTTCCTCCCACTCCAG | 71 |
|  | Reverse | TCACATGTGCGACAGGGGCAG | 73 |
| *mKlf4* | Forward | CACCATGGACCCGGGCGTGGCTGCCAGAAA | 84 |
|  | Reverse | TTAGGCTGTTCTTTTCCGGGGCCACGA | 76 |
| *mRex1* | Forward | GAGTGCATCATACGAGGTGAG | 65 |
|  | Reverse | TCCAGAACCTGGCGAGAAAGG | 69 |
| *mEsrrb* | Forward | CGCAAGAGCTACGAGGACTG | 68 |
|  | Reverse | GGTAGCCAGAGGCAATGTCC | 68 |
| *mUtf1* | Forward | AGGTTGCTCCCCAGTCGTTG | 70 |
|  | Reverse | TGGTTCAGGGTCGACAGCTG | 70 |
| *mSall1* | Forward | CATCATGAATGACAGCGAGGG | 66 |
|  | Reverse | CCGTTCTCCTGGAGACTGTC | 67 |
| *mCdx2* | Forward | TGCTGCAGACGCTCAACCTC | 71 |
|  | Reverse | TCCACTCGCACAGGTTTCGC | 71 |
| *mHand1* | Forward | ACAGAGAGCATTAACAGCGCG | 68 |
|  | Reverse | ACGTCCATCAAGTAGGCGATG | 67 |
| *mEomes* | Forward | CTGCACAAATACCAACCGAGG | 66 |
|  | Reverse | GAAGGTCTGAGTCTTGGAAGG | 65 |
| *mSnail* | Forward | TAGGTCGCTCTGGCCAACAT | 69 |
|  | Reverse | CTGGAAGGTGAACTCCACACA | 67 |
| *mT* | Forward | CCAGCTGCGTCTACATCCAC | 68 |
|  | Reverse | AAGCAGTGGCTGGTGATCATG | 68 |
| *mGsc* | Forward | AGATGCTGCCCTACATGAACG | 67 |
|  | Reverse | TGCTCATCGGTGAAGATGGTG | 67 |
| *mLlgl2* | Forward | ACCCAGAACCCAGAACCTCT | 68 |
|  | Reverse | ATGACACGGGAGGTGAAGTC | 67 |
| *mGrb7* | Forward | GGTTTCATGGACGCATCTCT | 65 |
|  | Reverse | AGGTCTGTGAAACGGGTCTG | 67 |
| *mFgf5* | Forward | CATCGGTTTCCATCTGCAGATC | 66 |
|  | Reverse | CTCCTCGTATTCCTACAATCCC | 64 |
| *mSox1* | Forward | CATGAACGCCTTCATGGTGTGG | 69 |
|  | Reverse | TCGGACATGACCTTCCACTCG | 69 |
| *mOtx2* | Forward | GGAAGTGAGTTCAGAGAGTGG | 65 |
|  | Reverse | TCCAGATAGACACTGGAGCAC | 66 |
| *mSox17* | Forward | CCAGATCTGCACAACGCAGAG | 69 |
|  | Reverse | TGGTCCTGCATATGCTGCACG | 71 |
| *mGata4* | Forward | ACTTCTCAGAAGGCAGAGAGTG | 66 |
|  | Reverse | GGTTGATGCCGTTCATCTTGTG | 67 |
| *mGata6* | Forward | ATCCAGACGCCACTGTGGAGA | 71 |
|  | Reverse | CTGAGGCCATTCATCTTGCTG | 66 |
| *mGAPDH* | Forward | CATCACCATCTTCCAGGAGC | 65 |
|  | Reverse | GCTGTAGCCGTATTCATTGTC | 63 |
